# Quantum chemical “Aufbau” principles: how to estimate the shape of highly flexible (bio-)polymers? A recursively extendable “chemion picture” of Euler-Hückel-type

**DOI:** 10.1007/s00894-023-05807-x

**Published:** 2024-01-24

**Authors:** Wolfhard H. G. Koch

**Affiliations:** 1 Facultad de Estudios Superiores Zaragoza, Universidad Nacional Autónoma de México, Mexico City, Mexico; 2 Institut für Physikalische und Theoretische Chemie, Universität Tübingen, Tübingen, Germany

**Keywords:** Irreducible Euler-Hückel vertex orbitals, Proportional nuclear charges, Chemionic separations, Vertex condensations, Recursively extendable “Aufbau” principles (REAP) and polymerizations

## Abstract

**Abstract:**

An outline is given of how to split the n-dimensional space of torsion angles occurring in flexible (bio-)polymers (like alkanes, nucleic acids, or proteins, for instance) into n one-dimensional potential curves. Forthcoming applications will focus on the “protein folding problem,” beginning with polyglycine.

**Context:**

In accordance with Euler’s topology rules, molecules are considered to be composed of “vertices” (atoms, ligands, bonding sites, functional groups, and bigger fragments). Following Hückel, each vertex is represented by only one basis function. Starting from the “monofocal” hydrids $\text {CH}_{4}$, $\text {NH}_{3}$, $\text {OH}_{2}$, FH, and $\text {SiH}_{4}$, $\text {PH}_{3}$, $\text {SH}_{2}$, ClH as anchor units, “chemionic” Hamiltonians (of individual “chemion ensembles” and proportional nuclear charges) are constructed recursively, together with an appropriate basis set for the first five (normal) alkanes and some related oligomers like primary alcohols, alkyl amines, and alkyl chlorides.

**Methods:**

Standard methods (“Restricted Hartree-Fock RHF” and “Full Configuration Interaction FCI”) are used to solve the various stationary Schrödinger equations. Two software packages are indispensable: “SMILES” for integral evaluations over Slater-type orbitals (STO), and “Numerical Recipes” for matrix diagonalizations and inversions. While managing with only two-center repulsion integrals, “implicit multi-center integrations” lead us to the non-empirical fundament of Hoffmann’s “Extended-Hückel Theory.”

## Introduction

Different from the physical view with electrons and nuclei as basic particles, chemists consider molecules to be built-up of $\#vch$ “valence chemions” localized at $\#vtx$ “vertices” [1–4]. Because vertex (sub)sets mostly are sharing four “chemion pairs” ($\#chp=\#vch/2$), they locally show a tetrahedral geometry (van’t Hoff [5] and Le Bel [6]). Keeping in mind this “octet rule” [7], bonding topologies of singly bound molecules can be graphically illustrated by Lewis’ [8–10] and Langmuir’s [11] formula language, commonly used in textbooks of organic and biochemistry.

Instead of saying “electron,” we are going to use the term “chemion” for mainly two reasons:

$$\circ $$
Due to their localization, chemions are characterized by an “*ensemble* individuality,” which severely violates the indistinguishability postulate of Schrödinger [12, 13] and Pauli [14].
$$\circ $$
Speaking of “valence chemions,” we claim the separability of the “valence shell” from its “atomic core” [15–17]. Later, we will even dare to distinguish “valence ligand chemions” and “valence bond chemions,” which dominantly govern the molecular shape due to their responsibility for interfragmental torsions.

Such classifications emphasize the fact that physicists and chemists sometimes look at the same molecular object from different points of view. The questions “Was sind Elektronen?” and “Kann Chemie auf Physik reduziert werden?” are thoroughly discussed in articles and books of Hans Primas and Ulrich Müller-Herold [18–25]. In this context, let us just mention another example of an extraordinary view: Topological properties of the charge density lead Bader to his quantum theoretical picture entitled “Atoms in Molecules” [26–30].

A priori predictions of molecular bond lengths, bond angles, dihedral and torsion angles are rather sophisticated [31–34]. “In *ab initio* electronic-structure calculations, approximate solutions are obtained to the molecular Schrödinger equation. The errors made in such calculations arise from the truncation of the one-electron space... and from the approximate treatment of the *N*-electron space...” [35]. If this is so, we cannot expect high-quality predictions of geometric parameters like bond lengths and bond angles. Focussing on highly flexible organic polymers (like alkane chains, proteins, or nucleic acids), their “secondary structure” is mostly determined by low-barrier rotations around single bonds [36, 37].

The quantum chemical picture to be outlined below is irreducible [38, 39]: being based on standard theories (like “Restricted Hartree-Fock” [40, 41] and “Full Configuration Interaction” [43]) to solve the time-independent Schrödinger equation [44]

1 $$\begin{aligned} \mathcal {H}\Psi _0=\Psi _0\cdot \mathcal {E}_0 \end{aligned}$$

it manages with an irreducible basis set of spherical “Slater-type orbitals” (STO) [45] of only the valence shell [46, 47]. Hence, it draws a more symbolic than quantitative sketch in the spirit of Leonhard Euler (1707–1783) [2–4] and Erich Hückel (1896–1980) [48–52]. Using standard “vertex”-geometries, we hope to get an idea of pure topological effects, which arise apart from a more sophisticated quantum theoretical treatment [53].

Next, we shall try to establish some “Quantum Chemical *Aufbau* Principles” [54, 55]. Introducing “vertex condensations” of Roothaan’s Fock-matrix expression, we are prepared to recursively construct growing diagonal elements from precomputed fragments. A successively augmented construction set will serve us to approximately build-up higher elements from lower units. Precomputed quantities thus can be readily taken from an external storage device, yielding a “Recursively Extendable Chemion Picture” [38, 39].

Similar to the atomic “*Aufbau* Principles” of Niels Bohr [56, 57] and Friedrich Hund [58, 59], we want to bring some order into quantum chemical descriptions of the molecular world. Therefore, we close the present paper with an outlook to straightforward generalizations, which might help us to estimate the shape of even macromolecular chains, particularly those of biochemical interest. If it becomes possible one day, to substitute our model assumptions by more realistic geometries, appropriate orbital exponents, and above all more complex fragments (including doubly bound groups like phenyl, carbonyl, and the peptide bond), this might be an important step towards a quantum chemical description of the “protein folding problem” [60].

It is one of the basic insights of chemical taxonomy, that molecules can be ordered into compound classes. Due to the recursive nature of our “Chemionic Hamiltonians,” classifications of molecular families become evident [61], this time also quantum chemically [62, 63].

During the recent decades, considerable progress has been made in our science. However, the topic “explicitly correlated coupled cluster calculations,” for instance, is beyond the scope of our article. Focusing on standard orbital theories with linear expansions, our main tools are “Restricted Hartree-Fock (RHF)” and “Full Configuration Interaction (FCI).”

The article has four chapters: 1. The Chemical Model; 2. Solution Methods; 3. Compound Classes; 4. Outlook and Conclusions. Appendices: Anchor Data and First Results.

## The chemical model

To begin with less complex examples, our paper focuses on polymerized organic chain molecules (normal, iso- and neo-alkanes; primary, secondary, and tertiary amines or alcohols, for instance). From an even more exposed viewpoint, our goal is the geometry prediction of biopolymers (like nucleic acids or proteins) with all their sensitive interactions (among them interfragmental torsions and hydrogen bonds). Always keeping in mind these background intentions, our present paper starts with some general considerations.

Based on the Born-Oppenheimer assumption [44], molecular orbital theories, in general, have to specify mainly three items:

$$\circ $$
The number of chemions under consideration
$$\circ $$
Position and charge of each nucleus
$$\circ $$
The basis set to be used

The quantum chemical picture to be drawn in this paper is summarized through the following headlines:

1.1. Euler Topologies
1.2. Recursive Polymerizations
1.3. Proportional Nuclear Charges
1.4. Chemionic Hamiltonians
1.5. Vertex Orbitals of Euler-Hückel-Type
1.5.1. Anchor Orbitals, their Exponents and Locations
1.5.2. Anchor Integrals
1.5.3. Recursion Orbitals and Integrals

### Euler topologies

“Vertices” can be synonymously understood as “bonding sites”: central atoms, hydrogen ligands, and “lone pair positions (love).” Beginning with the eight “monofocals” methane, ammonia, water, and hydrogen fluoride, as well as silane, phosphine, hydrogen sulfide, and hydrogen chloride

$$\begin{aligned}  &   {\textrm{H}}{-}{\textrm{C}}{\equiv }{\textrm{H}}_{3} \quad {\textrm{H}}{-}{\underline{\textrm{N}}}={\textrm{H}}_{2} \quad {\textrm{H}}{-}{\overline{\underline{\textrm{O}}}}{-}{\textrm{H}} \quad {\textrm{H}}{-}{\overline{\underline{\textrm{F}}}}|\nonumber \\  &   {\textrm{H}}{-}{\text {Si}}{\equiv }{\textrm{H}}_{3} \quad {\textrm{H}}{-}{{\underline{\textrm{P}}}}{=}{\textrm{H}}_{2} \quad {\textrm{H}}{-}{\overline{\underline{\textrm{S}}}}{-}{\textrm{H}} \quad {\textrm{H}}{-}{\overline{\underline{\text {Cl}}}}| \end{aligned}$$

we can easily see, that they all confirm the Eulerian topology rule [2–4]:

2 $$\begin{aligned} \underbrace{ \#vtx }_{=5} = \underbrace{ \#chp }_{=4} +1 \end{aligned}$$

Inspecting the Lewis-Langmuir formulae of more complex molecular frameworks, we find that the validity of this rule will always hold for “bifocals” (like ethane, methylamine, methanol, for instance), “trifocals” (like propane, ethylamine, ethanol $\dots$), and all the other “polyfocals” of such kind:

3 $$\begin{aligned}  &   {\textrm{H}}{-}({{{\textrm{C}{=}\textrm{H}_{2}}}}{\overset{\tau }{-}})_{\textrm{n}}{{{\textrm{C}}}}{\equiv }{\textrm{H}}_{3} \quad {\textrm{H}}{-}({{{\textrm{C}{=}\textrm{H}_{2}}}}{\overset{\tau }{-}})_{\textrm{n}}{{\underline{\textrm{N}}}}{=}{\textrm{H}}_{2} \nonumber \\  &   {\textrm{H}}{-}({{{\textrm{C}{=}\textrm{H}_{2}}}}{\overset{\tau }{-}})_{\textrm{n}}{\overline{\underline{\textrm{O}}}}{-}{\textrm{H}} \quad {\textrm{H}}{-}({{{\textrm{C}{=}\textrm{H}_{2}}}}{\overset{\tau }{-}})_{\textrm{n}}{\overline{\underline{\textrm{F}}}}|\nonumber \\  &   {\textrm{H}}{-}({{{\textrm{C}{=}\textrm{H}_{2}}}}{\overset{\tau }{-}})_{\textrm{n}}{{{\text {Si}}}}{\equiv }{\textrm{H}}_{3} \quad {\textrm{H}}{-}({{{\textrm{C}{=}\textrm{H}_{2}}}}{\overset{\tau }{-}})_{\textrm{n}}{{\underline{\textrm{P}}}}{=}{\textrm{H}}_{2} \nonumber \\  &   {\textrm{H}}{-}({{{\textrm{C}{=}\textrm{H}_{2}}}}{\overset{\tau }{-}})_{\textrm{n}}{\overline{\underline{\textrm{S}}}}{-}{\textrm{H}} \quad {\textrm{H}}{-}({{{\textrm{C}{=}\textrm{H}_{2}}}}{\overset{\tau }{-}})_{\textrm{n}}{\overline{\underline{\text {Cl}}}}|\nonumber \\  &   \underbrace{\#vtx}_{=3\textrm{n}+5}=\underbrace{\#chp}_{=3\textrm{n}+4}+1 \end{aligned}$$

It even remains valid for a chain of “functional groups” like H, $\text {CH}_{2}$, or $\text {CH}_{3}$, $\text {NH}_{2}$, OH, F, $\text {SiH}_{3}$, $\text {PH}_{3}$, $\text {SH}_{2}$, and Cl, the formulae of which do not take notice any more of their internal chemionic structure:

4 $$\begin{aligned}  &   {\textrm{H}}{-}({\text {CH}_{2}}{\overset{\tau }{-}})_{\textrm{n}}{\textrm{CH}}_{3} \quad {\textrm{H}}{-}({\text {CH}_{2}}{\overset{\tau }{-}})_{\textrm{n}}{\textrm{NH}}_{2} \nonumber \\  &   {\textrm{H}}{-}({\text {CH}_{2}}{\overset{\tau }{-}})_{\textrm{n}}{\textrm{OH}} \quad {\textrm{H}}{-}({\text {CH}_{2}}{\overset{\tau }{-}})_{\textrm{n}}{\textrm{F}}\nonumber \\  &   {\textrm{H}}{-}({\text {CH}_{2}}{\overset{\tau }{-}})_{\textrm{n}}{\text {SiH}}_{3} \quad {\textrm{H}}{-}({\text {CH}_{2}}{\overset{\tau }{-}})_{\textrm{n}}{\textrm{PH}}_{2} \nonumber \\  &   {\textrm{H}}{-}({\text {CH}_{2}}{\overset{\tau }{-}})_{\textrm{n}}{\textrm{SH}} \quad {\textrm{H}}{-}({\text {CH}_{2}}{\overset{\tau }{-}})_{\textrm{n}}{\text {Cl}}\nonumber \\  &   \underbrace{\#vtx}_{=\textrm{n} + 2}=\underbrace{\#chp}_{=\textrm{n} +1}+1 \end{aligned}$$

The geometry of such a polymer mainly depends on the n torsion angles $\tau$ [36, 37]. A non-gradient search for the global minimum of the corresponding n-dimensional “Born-Oppenheimer Energy Function (BOEF)” practically can only be done for n=1 or n=2 [64]. Therefore, we first have to answer the question of how to split a higher n-dimensional BOEF into n one-dimensional “potential curves.”

### Recursive polymerizations

Similar to a continuous polymerization process, which stepwise adds another monomer to its precursor, we consider a recursive chain growth. Taking the normal alkanes and primary amines and alcohols as an example, and using the convenient formula language (with X $= \text {CH}_{3}$, $\text {NH}_{2}$, OH) [8–11], it can be written down as in Table 1.

**Table 1:** Recursive polymerizations ($\textrm{X}=\text {CH}_{3},\text {NH}_{2},\text {OH}$)

	${\underbrace{ \textrm{H}_{3}\textrm{C}{\overset{\tau _{0}}{-}}(\text {CH}_{2})_{0} \textrm{X}}_{\textrm{H}_{3}\textrm{C}{\overset{\tau _{0}}{-}}\textrm{X}}}$	${\underbrace{ {\textrm{H}_{3}\textrm{C}}{\overset{\tau _1}{-}}({\text {CH}_{2}})_{1} {\textrm{X}} }_{\textrm{H}_{3}\textrm{C}{\overset{\tau _1}{-}}\text {CH}_{2}\textrm{X}}}$	${\underbrace{ {\textrm{H}_{3}\textrm{C}}{\overset{\tau _{2}}{-}}({\text {CH}_{2}})_{2} {\textrm{X}} }_{\textrm{H}_{3}\textrm{C}{\overset{\tau _2}{-}}\textrm{C}_{2}\textrm{H}_{4}\textrm{X}}}$	${\underbrace{ {\textrm{H}_{3}\textrm{C}}{\overset{\tau _{3}}{-}}({\text {CH}_{2}})_{3} {\textrm{X}} }_{\textrm{H}_{3}\textrm{C}\overset{\tau _3}{-}{\textrm{C}_{3}\textrm{H}_{6}\textrm{X}}}}$	...
n	0	1	2	3	...
$\#chp$	1	1	1	1	...
$\#vtx$	2	2	2	2	...
$\#nuc$(1)	4	4	4	4	...
$\#nuc$(2)	4, 3, 2	7, 6, 5	10, 9, 8	13, 12, 11	...

For each n, the numbers $\#chp$, $\#vtx$, and $\#nuc(1)$ remain constant; only the number $\#nuc(2)$ (i.e., the nuclei of the second vertex) augments steadily. Due to successive “vertex condensations” ${\textrm{X}}\rightarrow {\text {CH}_{2}\textrm{X}}\rightarrow {\textrm{C}_{2}\textrm{H}_{4}\textrm{X}}\rightarrow {\textrm{C}_{3}\textrm{H}_{6}\textrm{X}}\rightarrow \dots$, only one torsion angle $\tau _{\textrm{n}}$ has to be varied. For symmetry reasons, few of such variations may show some “degeneracy” [65–68].

### Proportional nuclear charges

In an electrically neutral molecule of Eqs. 2 and 3, the total chemion charge $2\#chp$ corresponds to the sum of “valence charges” ${\textrm{Z}}_{C}^{val}$ of all the nuclei (i.e., the column index of Mendelejew’s and Meyer’s “Periodic Table” [69–71]). Inspecting Table 1, however, $2\#chp{=}2$ chemion charges must be distributed among the $\#nuc$ nuclei under consideration.

In order to guarantee a proportional set of positive point charges for any chemionic *ensemble* $\epsilon$, we deal with “proportional valence charges” of the different nuclei [72, 73]:

5 $$\begin{aligned} {\textrm{Z}}^{prop}_{\epsilon C} \overset{~\text {def}~}{= } {\textrm{Z}}^{val}_C \cdot 2\#chp(\epsilon ) / \left( {\sum }_{D=1}^{\#nuc} {\textrm{Z}}^{val}_{D}\right) \quad ;\quad C=1,\dots ,\#nuc \end{aligned}$$

The nuclear repulsion energy with respect to any chemionic *ensemble* $\epsilon$ then reads as follows:

6 $$\begin{aligned} {\mathcal {E}}^{nucl}_{\epsilon } = \sum _{A=1}^{\#nuc} \sum _{B>A}^{\#nuc} { {\textrm{Z}}^{prop}_{\epsilon A} {\textrm{Z}}^{prop}_{\epsilon B} \over |{\textbf{R}}_{A}-{\textbf{R}}_{B}| } \end{aligned}$$

### Chemionic Hamiltonians

For a given chemionic *ensemble* $\epsilon$, the Born-Oppenheimer Hamiltonian reads as follows:

7 $$\begin{aligned}  &   {\mathcal {H}}_{\epsilon } \overset{\text {def}}{= } \sum _{i=1}^{\underset{(\epsilon )}{2\#chp}} \left\{ {\Delta ({{\textbf {r}}}_{i})\over -2}\!-\!\sum _{C=1}^{\#nuc}{{\textrm{Z}}^{prop}_{\epsilon C}\over |{{\textbf {r}}}_i\!-\!{{\textbf {R}}}_{C}|}\!+\!\sum _{j>i}^{\underset{(\epsilon )}{2\#chp}}{1\over |{{\textbf {r}}}_{i} \!-\! {{\textbf {r}}}_{j} |}\right\} ;\nonumber \\  &   {\Delta ({{\textbf {r}}}_{i})}\overset{\text {def}}{= }{\partial ^{2}\over \partial x_{i}^{2}}{+}{\partial ^{2}\over \partial y_{i}^2}{+}{\partial ^{2}\over \partial z_{i}^{2}} \end{aligned}$$

where $\Delta ({{\textbf {r}}}_{i})$ is the Laplace operator in Cartesian coordinates.

The components of the ground state energy

8 $$\begin{aligned} \mathcal {E}_0=\mathcal {E}^{nucl}_\epsilon +\mathcal {E}^{chem}_\epsilon \end{aligned}$$

- $\circ$ are the nuclear Born-Oppenheimer repulsion $\mathcal {E}^{nucl}_{\epsilon }$ of Eq. 6,
- $\circ$ and the chemionic part $\mathcal {E}^{chem}_{\epsilon }$, which will be identified with the lowest “Full Configuration Interaction” energy (FCI) of singlet or triplet multiplicity:
  9 $$\begin{aligned} \mathcal {E}^{chem}_{\epsilon } = {\left\{ \begin{array}{ll} \! {{~}^1}{\textbf{E}}_{1}^{\text {FCI}} \quad \text {if} {{~}^{1}} {\textbf{E}}_{1}^{\text {FCI}} \le \! {{~}^{3}}{\textbf{E}}_{1}^{\text {FCI}}\\ \! {{~}^3}{{\textbf {E}}}_{1}^{\text {FCI}} \quad \text {else} \end{array}\right. } \end{aligned}$$

### Vertex orbitals of Euler-Hückel-type

From Hückel, we adopt the idea that each Eulerian “anchor vertex” *V* is represented by only one basis orbital ${\textrm{n}}_{V}^{val}\textrm{s}$ of spherical symmetry [48–52], also for any “lone vertex (love).” The orbital type is specified through the following [70, 71]:

10 $$\begin{aligned} {\textrm{n}}^{val}_{V}= {\left\{ \begin{array}{ll}{ 1 \quad {\text {if}}\, V = {\textrm{H}} }\\ 2 \quad {\text {if}}\, V = \text { C, N, O,~F~or~their~``love''s} \\ 3 \quad {\text {if}}\, V = \text { Si, P, S,~Cl~or~their~``love''s} \end{array}\right. } \end{aligned}$$

which is the row index of the “Periodic Table” [70, 71].

#### Anchor orbitals, their exponents and locations

Orbital exponents are considered to be given by the ratio

11 $$\begin{aligned} \zeta _{V}:= {\textrm{Z}^{val}_{V}/{\textrm{n}}^{val}_{V}} \end{aligned}$$

Alternatively, we might follow the recommendations of Slater’s rules, which also incorporate an estimated “screening” [74–76]. The best thing to do, however, would be an exponent optimization by means of a non-linear variation procedure [77] (see Appendix A.3). Nevertheless, we like to stress that we aim at a more symbolic approach in the spirit of Erich Hückel [48–52].

In the tradition of van’t Hoff [5] and Le Bel [6], we consider mono-, bi-, and poly-tetrahedral model geometries (see Appendix A.1) with the following features:

$$\circ $$
Bond lengths $\lambda$ according to the Appendix A.2 [72, 73]
$$\circ $$
One unique tetrahedron bond angle $\theta := \arccos (-{1\over 3})~;$ $\theta ^{\circ }$ $= \theta \cdot {180^{\circ }\over \pi } \approx 109.471^{\circ }$
$$\circ $$
One unique dihedral angle $\delta :={2\pi \over 3}~;$ $\delta ^{\circ } = \delta \cdot {180^{\circ }\over \pi } = 120^{\circ }$
$$\circ $$
The full range of torsion angles $\tau$: $0\le \tau < 2\pi ~;$ $0^{\circ }\le \tau ^{\circ } =\tau \cdot {180^{\circ }\over \pi } < 360^{\circ }$

Due to the indistinguishability of the $2\#chp$ chemions within any “chemionic *ensemble*” $\epsilon$, we can take $\textbf{r}_1$ as a dummy position. $\phi _{m}\equiv \phi (\zeta _{m},\textbf{r}_1- \textbf{R}_{m})$ then stands for the *m*th “Euler-Hückel Vertex Orbital (EHVO)” located at $\textbf{R}_{m}$ with orbital exponent $\zeta _{m}$. Remember that all EHVO are of s-type [46, 47].

In our model, hence, “lone vertices (love)” are represented by a n$^{val}$s-function, localized at one of the peripheric tetrahedron positions; for the distance from their center, we adopt the Bohr radius $(\textrm{B} \approx 0.529177249~{\text{\AA }})$. As an artifact, EHVO bases produce “rotational barriers” also for axial symmetric halogen compounds like $\text {F}_{2}$, $\text {Cl}_{2}$, and FCl.

#### Anchor integrals

Quantum chemical calculations, in general, have to deal with at least four types of integrals:

$$\circ $$
“Overlap integrals” $$S_{(m.n)}^{(0)}$$
$$\circ $$
“Kinetic energy integrals” $K_{(m.n)}^{(0)}$
$$\circ $$
“Attraction energy integrals” $U_{(m.n.C)}^{(0)}$
$$\circ $$
The two-chemion “repulsion energy integrals” $R_{(m.n|p.q)}^{(0)}$

For the one- and two-chemion anchor integrals EHVI over EHVO, we use the following notations:

12 $$\begin{aligned} S_{(m.n)}^{(0)}= &   \int \phi _{m}(\textbf{r}_{1})\phi _{n}(\textbf{r}_{1})d\textbf{r}_{1}\nonumber \\ K_{(m.n)}^{(0)}= &   \int \phi _{m}(\textbf{r}_{1})\left\{ {\Delta (\textbf{r}_{1})\over -2}~\phi _{n}({\textbf{r}}_{1})\right\} d\textbf{r}_{1} \nonumber \\ U_{(m.n.C)}^{(0)}= &   {\int }{\phi _{m}(\textbf{r}_{1})\phi _{n}(\textbf{r}_{1}) | \textbf{r}_{1}-\textbf{R}_{C}|}^{-1}d\textbf{r}_{1}\nonumber \\ R_{(m.n|p.q)}^{(0)}= &   \int \int \phi _{m}(\textbf{r}_{1})\phi _{n}(\textbf{r}_{1})\left\{ |{\textbf{r}}_{1}-\textbf{r}_{2} |^{-1}\right\} \nonumber \\    &   \times \phi _{p}(\textbf{r}_{2}) \phi _{q}(\textbf{r}_{2})d\textbf{r}_{1}d\textbf{r}_{2}\nonumber \\ {m,n},{p,q}= &   1,\dots ,\#vtx ;\displaystyle {C}=1,\dots ,{\#nuc} \end{aligned}$$

Numerical evaluations are available by the SMILES package of FORTRAN routines, for instance [78–80].

Summing up the charge-weighted “attraction energy integrals,” we arrive at the $\epsilon$-dependent “attraction energy matrix” ${V}^{(0)}_{\epsilon (m.n)}$; subtracted from $K_{(m.n)}^{(0)}$, it yields the matrix $H_{\epsilon (m.n)}^{(0)}$, which represents the “one-chemion energy”:

13 $$\begin{aligned}  &   {V}^{(0)}_{\epsilon (m.n)} \overset{\text {def}}{= } {\sum }_{C=1}^{\#nuc} {\textrm{Z}}^{prop}_{\epsilon C} {U}^{(0)}_{(m.n.C)} \quad ~;~ \nonumber \\  &   {H}^{(0)}_{\epsilon (m.n)} \overset{\text {def}}{ = } {K}^{(0)}_{(m.n)} - {V}^{(0) }_{\epsilon (m.n)} \end{aligned}$$

#### Recursion orbitals and integrals

Now let us return to the simplified Lewis/Langmuir-formulas of Table 1, which promise the successive “*Aufbau*” of macromolecular chains from smaller units:

14 $$\begin{aligned} \begin{array}{ccc} \overbrace{\underbrace{\textrm{H}_{3}\textrm{C}}_{\alpha \alpha }\overset{\tau _\textrm{n}}{-}\underbrace{({\text {CH}_{2}})_{\textrm{n}}{\text {XV}_{3}}}_{\beta \beta ^{(\textrm{n})}}}^{\alpha \beta ^{(\textrm{n})}} & \quad ;\quad {\begin{array}{ccc} {\#chp=1}\\ {\#vtx=2}\end{array}}&\quad ; \quad \textrm{n}=0,1,2,3,\dots \end{array} \end{aligned}$$

As we can see, each prolongation step n is composed of two “diagonal” and one “off-diagonal” contribution ($\alpha \alpha$, $\beta \beta ^{(\text {n})}$, and $\alpha \beta ^{(\text {n})}$, respectively).

In order to construct two basis functions for all the chain molecules with $\#chp=1$, we define the following set of normalized “Recursion Orbitals”

15 $$\begin{aligned} \begin{matrix} \phi ^{(\textrm{n})}_{\omega }(\textbf{r}_1)&  \!\overset{\text {def}}{ = }\!{\mathcal {N}}^{(\textrm{n}-1)}_{\omega }\textstyle \sum _{p=1}^{\#vtx(\omega )}\phi ^{(\textrm{n}-1)}_{p}({\textbf {r}}_{1}) \\ \mathcal {N}^{(\textrm{n}-1)}_{\omega } & \overset{\text {def}}{ = }\textstyle \left\{ \sum _{p=1}^{\#vtx(\omega )}\sum _{q=1}^{\#vtx(\omega )}{S}^{(\textrm{n}-1)}_{(p.q)}\right\} ^{-\frac{1}{2}} \end{matrix} \begin{array}{l} \!\quad \! {;\! \quad \!\begin{array}{l}\mathrm{n\!=\!0,1,2,3,\dots }\\ {\omega =1,2}\end{array}} \end{array} \end{aligned}$$

With them, we arrive at

$$\circ $$
“recursive overlap integrals” ${S}^{(\textrm{n})}_{(\alpha .\beta )}$
$$\circ $$
“recursive kinetic integrals” ${K}^{(\textrm{n})}_{(\alpha .\beta )}$
$$\circ $$
“recursive attraction integrals” ${U}^{(\textrm{n})}_{(\alpha .\beta .C )}$
$$\circ $$
and “recursive repulsion integrals” ${R}^{(\textrm{n})}_{(\alpha .\beta |\gamma .\delta )}$

They read as follows:

16 $$\begin{aligned} {S}^{(\textrm{n})}_{(\alpha .\beta )}&\overset{~\text {def}}{= }{\mathcal {N}}^{(\textrm{n}-1)}_{\alpha }{\mathcal {N}}^{(\textrm{n}-1)}_{\beta }\displaystyle \sum _{m=1}^{\underset{(\alpha )}{\#vtx}}\sum _{n=1}^{\underset{(\beta )}{\#vtx}}{S}^{(\textrm{n}-1)}_{(m.n)} \nonumber \\ {K}^{(\textrm{n})}_{(\alpha .\beta )}&\overset{~\text {def}}{= }{\mathcal {N}}^{(\textrm{n}-1)}_{\alpha }{\mathcal {N}}^{(\textrm{n}-1)}_{\beta }\displaystyle \sum _{m=1}^{\underset{(\alpha )}{\#vtx}}\sum _{n=1}^{\underset{(\beta )}{\#vtx}}{K}^{(\textrm{n}-1)}_{(m.n)}\nonumber \\ {U}^{(\textrm{n})}_{(\alpha .\beta .C)}&\overset{~\textrm{def}}{= }{\mathcal {N}}^{(\textrm{n}-1)}_{\alpha }{\mathcal {N}}^{(\textrm{n}-1)}_{\beta }\displaystyle \sum _{m=1}^{\underset{(\alpha )}{\#vtx}}\sum _{n=1}^{\underset{(\beta )}{\#vtx}}{U}^{(\textrm{n}-1)}_{(m.n.C)}\nonumber \\ {R}^{(\textrm{n})}_{(\alpha .\beta |\gamma .\delta )}&\overset{~\text {def}}{= }_{{\mathcal {N}}^{(\textrm{n}-1)}_{\gamma }{\mathcal {N}}^{(\textrm{n}-1)}_{\delta }}^{~{\mathcal {N}}^{(\textrm{n}-1)}_{\alpha }{\mathcal {N}}^{(\textrm{n}-1)}_{\beta }}\displaystyle \sum _{m=1}^{\underset{(\alpha )}{\#vtx}}\sum _{n=1}^{\underset{(\beta )}{\#vtx}}\sum _{p=1}^{\underset{(\gamma )}{\#vtx}}\sum _{q=1}^{\underset{(\delta )}{\#vtx}}{R}^{(\mathrm n-1)}_{(m.n|p.q)}\nonumber \\ \alpha ,&\beta , \gamma ,\delta =1,2; C=1,\dots ,{\#nuc} \end{aligned}$$

The “recursive attraction matrix” and the “recursive one-chemion matrix” then read as follows:

17 $$\begin{aligned}  &   {V}^{(\textrm{n})}_{\epsilon (\alpha .\beta )} \overset{\text {def}}{= }{\sum }_{C=1}^{\#nuc}{\textrm{Z}}^{prop}_{\epsilon C}{U}^{(\textrm{n}) }_{(\alpha .\beta .C)} \quad ~;~\nonumber \\  &   {H}^{(\textrm{n})}_{\epsilon (\alpha .\beta )}\overset{\text {def}}{= }{K}^{(\textrm{n})}_{(\alpha .\beta )}-{V}^{(\textrm{n})}_{\epsilon (\alpha .\beta )} \end{aligned}$$

Let us illustrate some recursion relations between the diagonal $\textbf{M}$-matrix elements of ethane, propane, normal butane, normal pentane etc., where $\textbf{M}\in \{\textbf{S},\textbf{K}\}$.

Starting from ${\text {HCH}_{2}}\rightarrow {M}^{(0)}_{(\alpha ,\alpha )}$ and ${\text {XV}_{3}}\rightarrow {M}^{(0)}_{(\beta ,\beta )}$18Recursion relations for the $\epsilon$-dependent matrices $\textbf{M}_\epsilon \in \{\textbf{V}_{\epsilon },{\textbf {H}}_{\epsilon }\}$ will be discussed below, in the context of Roothaan’s Fock matrix expressions.

## Solution methods

In order to approximately solve the time-independent Schrödinger Eq. 1, we start from an expansion of the molecular orbitals in terms of the vertex basis. In spite of the linear nature of this expansion, such “Roothaan-Hartree-Fock Orbitals (RHFO)” have to be determined iteratively. Expanding the wave function in terms of all the constructable singlet and triplet configurations, corresponding ground state energies are available by linear variations. Non-linear variations come in through an optimization of a few orbital exponents. The methodical chapter is subdivided into three sections:

2.1. Linear combinations of vertex orbitals
2.2. Self-consistent Roothaan-Hartree-Fock orbitals
2.3. Full configuration interaction

### Linear combinations of vertex orbitals

Molecular RHFO are linear combinations of the given EHVO:

19 $$\begin{aligned} \psi _{a}({\textbf {r}}_{1}) \overset{\text {def}}{ = }\sum _{m=1}^{\underset{(\epsilon )}{\#vtx}} C^{\text {RHF}}_{(m.a)}\phi ^{(\textrm{n})}_{m}(\textbf{r}_1) \qquad ;~ \begin{array}{l} \mathrm{n=0,1,2,3,}\ldots \\ a=1,\ldots ,\#vtx(\epsilon ) \end{array} \end{aligned}$$

For convenience, we skipped the chain length (n) on the left side.

With this expansion, molecular “Roothaan-Hartree-Fock Integrals (RHFI)” relate to the “Euler-Hückel Vertex Integrals (EHVI)” as follows:

20 $$\begin{aligned}&h_{\epsilon (ab)} =\sum _{\alpha =1}^{\underset{(\epsilon )}{\#vtx}} \sum _{\beta =1}^{\underset{(\epsilon )}{\#vtx}} C_{(\alpha .a)}^{\text {RHF}} C_{(\beta .b)}^{\text {RHF}} H_{\epsilon (\alpha .\beta )}^{(\text {n})} \end{aligned}$$

21 $$\begin{aligned} (ab|cd) \!=\!&\displaystyle \sum _{\alpha =1}^{\underset{(\epsilon )}{\#vtx}} \sum _{\beta =1}^{\underset{(\epsilon )}{\#vtx}} \sum _{\gamma =1}^{\underset{(\epsilon )}{\#vtx}} \sum _{\delta =1}^{\underset{(\epsilon )}{\#vtx}} C_{(\alpha .a)}^{\text {RHF}} C_{(\beta .b)}^{\text {RHF}} C_{(\gamma .c)}^{\text {RHF}} C_{(\delta .d)}^{\text {RHF}} R_{(\alpha .\beta |\gamma .\delta )}^{(\text {n})} \end{aligned}$$

For large basis sets, the transformation of Eq. 21, in particular, is quite time-consuming. Due to the irreducible Euler-Hückel expansion, however, its expense is moderate.

### Self-consistent Roothaan-Hartree-Fock orbitals

Roothaan’s expression of the Fock-matrix [40, 41] now reads as follows:

22 $$\begin{aligned} F^{(\textrm{n})}_{\epsilon (\alpha .\beta )}\overset{\mathrm{~def}}{= }\  &{\left\{ \begin{array}{ll} \mathcal {N}^{(\textrm{n}-1)}_{\alpha }\mathcal {N}^{(\textrm{n}-1)}_{\beta }\displaystyle \sum _{m=1}^{\underset{(\alpha )}{\#vtx}}\sum _{n=1}^{\underset{(\beta )}{\#vtx}}{F}^{(\textrm{n}-1)}_{\epsilon (m.n)} &  {\text {if~available}}\\ H_{\epsilon (\alpha .\beta )}^{(\textrm{n})}+W_{\epsilon (\alpha .\beta )}^{(\textrm{n})\text {cou}}-{1\over 2}W_{\epsilon (\alpha .\beta )}^{(\textrm{n})\text {exc}}&  \text {otherwise}\end{array}\right. }\nonumber \\ W_{\epsilon (\alpha .\beta )}^{(\textrm{n})\text {cou}}=&\sum _{\gamma =1}^{\underset{(\epsilon )}{\#vtx}}\sum _{\delta =1}^{\underset{(\epsilon )}{\#vtx}}R_{(\alpha .\beta |\gamma .\delta )}^{(\textrm{n})}D_{\epsilon (\gamma .\delta )}^{(\textrm{n})} \nonumber \\ W_{\epsilon (\alpha .\beta )}^{\text {(n)exc}}=&\sum _{\gamma =1}^{\underset{(\epsilon )}{\#vtx}}\sum _{\delta =1}^{\underset{(\epsilon )}{\#vtx}}{1\over 2}\left\{ R_{(\alpha .\gamma |\beta .\delta )}^{(\textrm{n})}+R_{(\alpha .\delta |\gamma .\beta )}^{(\textrm{n})}\right\} D_{\epsilon (\gamma .\delta )}^{(\textrm{n})}\nonumber \\ D_{\epsilon (\gamma .\delta )}^{(\textrm{n})}=&2{\sum }^{\#chp(\epsilon )}_{o=1}C_{(\gamma .o)}^{\text {RHF}}C_{(\delta .o)}^{\text {RHF}}\nonumber \\ {\textrm{n}}=0,1,2,&~3,\ldots ; \alpha , \beta =1,\ldots ,\#vtx(\epsilon ) \end{aligned}$$

$\textbf{D}_{\epsilon }^{(\textrm{n})}$ is the density matrix [40, 41] of the chemionic *ensemble* $\epsilon$.

Recursion relations for the matrices $\textbf{M}_{\epsilon }\in \{{\textbf{H}}_{\epsilon },\textbf{F}_{\epsilon }\}$ essentially are those of Eq. 18. This time, however, they additionly depend on the “diagonal” or “off-diagonal” *ensemble* parameter $\epsilon$, as indicated above.

Given both Fock and overlap matrices $\textbf{F}_{\epsilon }^{(\textrm{n})}$ and $\textbf{S}^{(\textrm{n})}$, respectively, the Roothaan equation

23 $$\begin{aligned} \textbf{F}^{(\textrm{n})}_{\epsilon }{\textbf{C}}^{\text {RHF}}=\textbf{S}^{(\textrm{n})}\textbf{C}^{\text {RHF}}\textbf{E}^{\text {RHF}} \quad ;\quad (\#vtx(\epsilon ) \times \#vtx(\epsilon )) \end{aligned}$$

can be solved by means of standard techniques [40–42]. Due to the density dependence of $\textbf{F}_{\epsilon }$, however, the coefficient matrix $\textbf{C}^{\text {RHF}}$ and the diagonal matrix $\textbf{E}^{\text {RHF}}$ of orbital energies have to be determined iteratively. Self-consistence of the density matrix has been achieved, if for successive iterations (*i*) and $(i-1)$ the following convergence criterion $\sigma {(i)}$ falls below a predefined threshold:

24 $$\begin{aligned}  &   \sigma {(i)} {~}\overset{\text {def}}{= } \left\{ (\#vtx(\epsilon ))^{-2} \textstyle \sum _{m,n=1}^{\#vtx(\epsilon )}\right. \nonumber \\  &   \qquad \qquad \quad \left. \left[ D_{\epsilon (m.n)}^\mathrm{(n)}{(i)} - D_{\epsilon (m.n)}^\mathrm{(n)}{(i-1)} \right] ^{2} \right\} ^{1\over 2} <10^{-4} \end{aligned}$$

### Full configuration interaction

Seeking for solutions of the time-independent Schrödinger Eq. 1, “Full Configuration Interaction (FCI)” “is the best we can do” [43]. Although being sparse, however, the so-called “minimal basis set” [40, 41] representations $\textbf{H}^{\text {FCI}}$ of many-chemion Hamiltonians are truly gigantic, even for rather small molecules [43].

Due to the irreducible nature of the chemionic vertex picture (with $\#vtx=\#chp+1$), on the other hand, the matrix dimensions $\#sing$ and $\#trip$ of its singlet block ^1^$\textbf{H}^{\text {FCI}}_{\epsilon }$ and triplet block ^3^$\textbf{H}^{\text {FCI}}_{\epsilon }$, respectively, are quite moderate:

25 $$\begin{aligned} \#sing =&\#vtx(\#vtx+1)/2=(\#chp+1)(\#chp+2)/2 \nonumber \\ \#trip =&\#vtx(\#vtx-1)/2=(\#chp+1)(\#chp)/2 \end{aligned}$$

The corresponding eigenvalue equations now read as follows:

26 $$\begin{aligned} \!{~}^{1}\textbf{H}^{\text {FCI}}_{\epsilon }~\!{~}^{1}\textbf{C}^{\text {FCI}}= &   \!{~}^{1}\textbf{C}^{\text {FCI}}~\!\!{~}^{1}\textbf{E}^{\text {FCI}}\nonumber \\ (\!{~}^{1}\textbf{C}^{\text {FCI}})^\dagger ~\!{~}^{1}{\textbf{H}}^{\text {FCI}}_{\epsilon }~\!{~}^{1}{\textbf{C}}^{\text {FCI}}= &   \!{~}^{1}\textbf{E}^{\text {FCI}} \quad ;\quad (\#sing\times \#sing) \end{aligned}$$

27 $$\begin{aligned} \!{~}^{3}\textbf{H}^{\text {FCI}}_{\epsilon }~\!{~}^{3}\textbf{C}^{\text {FCI}}= &   \!{~}^{3}\textbf{C}^{\text {FCI}}~\!{~}^{3}\textbf{E}^{\text {FCI}}\nonumber \\ (\!{~}^{3}\textbf{C}^{\text {FCI}})^\dagger ~\!{~}^{3}\textbf{H}^{\text {FCI}}_{\epsilon }~\!{~}^{3}\textbf{C}^{\text {FCI}}= &   \!{~}^{3}\textbf{E}^{\text {FCI}} \quad ;\quad (\#trip\times \#trip) \end{aligned}$$

Matrix elements may become zero due to group theoretical reasons, with respect to the spatial symmetry of the molecule [65–68]. Regarding the “spin operators” $\mathcal {S}^{2}$ and $\mathcal {S}_{z}$, matrix elements between states ${\!{~}^{(2{S}+1)}_{M_{S}} \Psi }$ with different quantum numbers $S\in \{0,1\}$ and $M_{S}$ (with integer values $-S\le M_{S}\le +S$) vanish [44]: triplet states thus neither mix with singlet states nor with other triplet states of different $M_{S}$.

For intermediately normalized wave functions [43], the singlet-type matrix elements (upper triangle) with non-vanishing expressions read as follows:

28 $$\begin{aligned} \langle ^{1}_{0}\Psi _{0} | \mathcal {H}_{\epsilon } |^{1}_{0}\Psi _{0}\rangle= &   \textstyle \sum _{m \ne r}^{\#vtx}\left\{ 2h_{_\epsilon (mm)}\right. \nonumber \\  &   \left. +\textstyle \sum _{n \ne r}^{\#vtx}\left[ 2J_{mn}-K_{mn}\right] \right\} {~}\overset{\text {def}}{= }\mathcal {E}_{0}^{\text {RHF}} \nonumber \\ \langle ^{1}_{0}\Psi _{aa}^{rr}| \mathcal {H}_{\epsilon } |^{1}_{0}\Psi _{aa}^{rr}\rangle= &   \mathcal {E}_{0}^{\text {RHF}}+2(\varepsilon _{r}-\varepsilon _{a})+J_{rr}\nonumber \\  &   +J_{aa}-4J_{ar}+2K_{ar} \nonumber \\ \langle ^{1}_{0}\Psi _{a}^{r}|\mathcal {H}_{\epsilon }|^{1}_{0}\Psi _{a}^{r}\rangle= &   \mathcal {E}_{0}^{\text {RHF}}+F_{rr}^{r}-F_{aa}^{r} - J_{ar}+2K_{ar}\nonumber \\ \langle ^{1}_{0}\Psi _{ab}^{rr}|\mathcal {H}_{\epsilon } |^{1}_{0}\Psi _{ab}^{rr}\rangle= &   \mathcal {E}_{0}^{\text {RHF}}+2\varepsilon _{r} - \varepsilon _{a} - \varepsilon _{b} + J_{rr}+J_{ab}\nonumber \\  &   -2J_{ar}-2J_{br}+K_{ab}+K_{ar}+K_{br}\nonumber \\ \langle ^{1}_{0}\Psi _{0}| \mathcal {H}_{\epsilon } |^{1}_{0}\Psi _{aa}^{rr}\rangle= &   + K_{ar}\nonumber \\ \langle ^{1}_{0}\Psi _{0}| \mathcal {H}_{\epsilon } |^{1}_{0}\Psi _{a}^{r}\rangle= &   + 2^{+{1\over 2}}F_{ar}^r{~}\overset{\text {RHF}}{=}0\nonumber \\ \langle ^{1}_{0}\Psi _{0}| \mathcal {H}_{\epsilon } |^{1}_{0}\Psi _{ab}^{rr}\rangle= &   + 2^{+{1\over 2}}(ar|br) \nonumber \\ \langle ^{1}_{0}\Psi _{aa}^{rr}| \mathcal {H}_{\epsilon } |^{1}_{0}\Psi _{a}^{r}\rangle= &   + 2^{+{1\over 2}}F_{ar}^{a} \nonumber \\ \langle ^{1}_{0}\Psi _{aa}^{rr}| \mathcal {H}_{\epsilon } |^{1}_{0}\Psi _{b}^{r}\rangle= &   - 2^{+{1\over 2}}(ab|ar) \nonumber \\ \langle ^{1}_{0}\Psi _{aa}^{rr}| \mathcal {H}_{\epsilon } |^{1}_{0}\Psi _{ab}^{rr}\rangle= &   -2^{+{1\over 2}}F_{ab}^{a} \nonumber \\ \langle ^{1}_{0}\Psi _{aa}^{rr}| \mathcal {H}_{\epsilon } |^{1}_{0}\Psi _{bc}^{rr}\rangle= &   +2^{+{1\over 2}}(ab|ac) \nonumber \\ \langle ^{1}_{0}\Psi _{a}^{r}| \mathcal {H}_{\epsilon } |^{1}_{0}\Psi _{b}^{r}\rangle= &   -F_{ab}^{r}-(ab|rr)+2(ar|rb) \nonumber \\ \langle ^{1}_{0}\Psi _{a}^{r}| \mathcal {H}_{\epsilon } |^{1}_{0}\Psi _{ab}^{rr}\rangle= &   +\{h_{\epsilon (br)}+\textstyle \sum _{n\ne a}^{\#vtx}(br|nn)\nonumber \\  &   +\sum _{n\ne r}^{\#vtx}\left[ (br|nn)-(bn|nr)\right] \}-(ab|ar) \nonumber \\ \langle ^{1}_{0}\Psi _{a}^{r}| \mathcal {H}_{\epsilon } |^{1}_{0}\Psi _{bc}^{rr}\rangle= &   -(ab|cr)-(ac|br) \nonumber \\ \langle ^{1}_{0}\Psi _{ab}^{rr}| \mathcal {H}_{\epsilon } |^{1}_{0}\Psi _{bc}^{rr}\rangle= &   -\{h_{\epsilon (ac)}+\textstyle \sum _{n\ne a}^{\#vtx}(ac|nn)\nonumber \\  &   +\sum _{n\ne b}^{\#vtx}\left[ (ac|nn)-(an|nc)\right] \}+(ab|bc) \nonumber \\ \langle ^{1}_{0}\Psi _{ab}^{rr}| \mathcal {H}_{\epsilon } |^{1}_{0}\Psi _{cd}^{rr}\rangle= &   +(ac|bd)+(ad|bc) \end{aligned}$$

where we used the following relations known from RHF theory [40, 41]:

29 $$\begin{aligned}&J_{ab}{~}\overset{\text {def}}{= }(aa|bb)\qquad ;\qquad K_{ab}{~}\overset{\text {def}}{= }(ab|ba) \nonumber \\&\varepsilon _{k}{~}\overset{\text {def}}{= }h_{\epsilon (kk)}+\textstyle \sum _{n \ne r}^{\#vtx}\left[ 2J_{kn}-K_{kn}\right] \nonumber \\&{F}_{kl}^{m}{~}\overset{\text {def}}{= }h_{\epsilon (kl)}+\textstyle \sum _{n \ne m}^{\#vtx}\left[ 2(kl|nn)-(kn|nl)\right] \end{aligned}$$

**Table 2:** Iso-chemionic compound classes

Family	Formula	*#chp*	*#vtx*	Vertices	*#sing*	*#trip*
Normal alkanes	$\textrm{R}_{\textrm{a}}{-}\text {CH}_{2}{-}\textrm{R}_{\textrm{b}}$	2	3	$\text {CH}_{2},\textrm{R}_{\textrm{a}},\textrm{R}_{\textrm{b}}$	6	3
Iso-alkanes	$$\begin{array}{l}{\textrm{R}_{\textrm{a}}{-}}\\ {\textrm{R}_\textrm{b}{-}} \end{array}\text {CH} {-}\textrm{R}_{\textrm{c}}$$	3	4	$\text {CH},\textrm{R}_{\textrm{a}},\textrm{R}_{\textrm{b}},\textrm{R}_{\textrm{c}}$	10	6
Neo-alkanes	$$ \begin{array}{l}{\textrm{R}_{\textrm{a}}{-}}\\ {\textrm{R}_{\textrm{b}}{-}}\end{array} \textrm{C} \begin{array}{l}{-}\textrm{R}_{\textrm{c}} \\ {-\textrm{R}_{\textrm{d}}} \end{array} $$	4	5	$\textrm{C},\textrm{R}_{\textrm{a}},\textrm{R}_{\textrm{b}},\textrm{R}_{\textrm{c}},\textrm{R}_{\textrm{d}}$	15	10
Prim. amines	$\textrm{R}_{\textrm{a}}{-}\text {CH}_{2}{-}\text {NH}_{\textrm{2}}$	2	3	$\text {CH}_{\textrm{2}},\textrm{R}_{\textrm{a}},\text {NH}_{2}$	6	3
Sec. amines	$$\begin{array}{l}{\textrm{R}_{\textrm{a}}{-}}\\ {\textrm{R}_{\textrm{b}}{-}}\end{array} \text {CH}{-}\text {NH}_{2}$$	3	4	$\text {CH},\textrm{R}_{\textrm{a}},\textrm{R}_{\textrm{b}},\text {NH}_{2}$	10	6
Tert. amines	$$\begin{array}{l}{\textrm{R}_{\textrm{a}}{-}}\\ {\textrm{R}_{\textrm{b}}{-}}\end{array}\textrm{C} \begin{array}{l}{-\textrm{R}_{\textrm{c}}}\\ {-\text {NH}}_{2} \end{array} $$	4	5	$\textrm{C},\textrm{R}_{\textrm{a}},\textrm{R}_{\textrm{b}},\textrm{R}_{\textrm{c}},\text {NH}_{2}$	15	10
Prim. alcohols	$\textrm{R}_{\textrm{a}}{-}\text {CH}_{2}{-}\text {OH}$	2	3	$\text {CH}_{2},\textrm{R}_{\textrm{a}},\text {OH}$	6	3
Sec. alcohols	$$\begin{array}{l}\textrm{R}_{\textrm{a}}{-}\\ {\textrm{R}_{\textrm{b}}{-}}\end{array}\text {CH}{-}\text {OH}$$	3	4	$\text {CH},\textrm{R}_{\textrm{a}},\textrm{R}_{\textrm{b}},\text {OH}$	10	6
Tert. alcohols	$$\begin{array}{l}{\textrm{R}_{\textrm{a}}{-}}\\ {\textrm{R}_{\textrm{b}}{-}} \end{array} \textrm{C} \begin{array}{l}{-\textrm{R}_{\textrm{c}}}\\ {-\text {OH}}\end{array}$$	4	5	$\textrm{C},\textrm{R}_{\textrm{a}},\textrm{R}_{\textrm{b}},\textrm{R}_{\textrm{c}},\text {OH}$	15	10
Prim. chlorides	$\textrm{R}_{\textrm{a}}{-}\text {CH}_{2}{-}\text {Cl}$	2	3	$\text {CH}_{2},\textrm{R}_{\textrm{a}},\text {Cl}$	6	3
Sec. chlorides	$$\begin{array}{l}{\textrm{R}_{\textrm{a}}{-}}\\ {\textrm{R}_{\textrm{b}}{-}} \end{array} \text {CH}{-}\text {Cl} $$	3	4	$\text {CH},\textrm{R}_{\textrm{a}},\textrm{R}_{\textrm{b}},\text {Cl}$	10	6
Tert. chlorides	$$ \begin{array}{l}{\textrm{R}_{\textrm{a}}{-}}\\ {\textrm{R}_{\textrm{b}}{-}}\end{array} \textrm{C}\begin{array}{l}-\textrm{R}_{\textrm{c}}\\ {-\text {Cl} }\end{array}$$	4	5	$\textrm{C},\textrm{R}_{\textrm{a}},\textrm{R}_{\textrm{b}},\textrm{R}_{\textrm{c}},\text {Cl}$	15	10

The triplet-type matrix elements (upper triangle) with non-vanishing expressions read as follows:

30 $$\begin{aligned}&\langle ^{3}_{0}\Psi _{a}^{r}|\mathcal {H}_{\epsilon } |^{3}_{0}\Psi _{a}^{r}\rangle =\langle ^{1}_{0}\Psi _{a}^{r}|\mathcal {H}_{\epsilon } |^{1}_{0}\Psi _{a}^{r}\rangle -4K_{ar} \nonumber \\&\langle ^{3}_{0}\Psi _{ab}^{rr}| \mathcal {H}_{\epsilon } |^{3}_{0}\Psi _{ab}^{rr}\rangle \!=\!\langle ^{1}_{0}\Psi _{ab}^{rr}| \mathcal {H}_{\epsilon } |^{1}_{0}\Psi _{ab}^{rr}\rangle \!-\!2K_{ab}\!-\!2K_{ar}\!-\!2K_{br} \nonumber \\&\langle ^{3}_{0}\Psi _{a}^{r}| \mathcal {H}_{\epsilon } |^{3}_{0}\Psi _{b}^{r}\rangle =\langle ^{1}_{0}\Psi _{a}^{r}| \mathcal {H}_{\epsilon }|^{1}_{0}\Psi _{b}^{r}\rangle -4(ar|rb) \nonumber \\&\langle ^{3}_{0}\Psi _{a}^{r}| \mathcal {H}_{\epsilon } |^{3}_{0}\Psi _{ab}^{rr}\rangle =\langle ^{1}_{0}\Psi _{a}^{r}| \mathcal {H}_{\epsilon } |^{1}_{0}\Psi _{ab}^{rr}\rangle +2(ab|ar) \nonumber \\&\langle ^{3}_{0}\Psi _{a}^{r}| \mathcal {H}_{\epsilon }|^{3}_{0}\Psi _{bc}^{rr}\rangle =\langle ^{1}_{0}\Psi _{a}^{r}| \mathcal {H}_{\epsilon } |^{1}_{0}\Psi _{bc}^{rr}\rangle +2(ac|br) \nonumber \\&\langle ^{3}_{0}\Psi _{ab}^{rr}| \mathcal {H}_{\epsilon } |^{3}_{0}\Psi _{bc}^{rr}\rangle =\langle ^{1}_{0}\Psi _{ab}^{rr}|\mathcal {H}_{\epsilon } |^{1}_{0}\Psi _{bc}^{rr}\rangle -2(ab|bc) \nonumber \\&\langle ^{3}_{0}\Psi _{ab}^{rr}| \mathcal {H}_{\epsilon } |^{3}_{0}\Psi _{cd}^{rr}\rangle =\langle ^{1}_{0} \Psi _{ab}^{rr}| \mathcal {H}_{\epsilon }|^{1}_{0}\Psi _{cd}^{rr}\rangle -2(ad|bc) \end{aligned}$$

Triplet states are triply degenerate:

31 $$\begin{aligned} \langle ^{3}_{0}\Psi | \mathcal {H}_{\epsilon }|^{3}_{0}\Psi \rangle =\langle ^{~\>3}_{+1}\Psi |\mathcal {H}_{\epsilon }|^{~\>3}_{+1}\Psi \rangle =\langle ^{~\>3}_{-1}\Psi | \mathcal {H}_{\epsilon } |^{~\>3}_{-1}\Psi \rangle \end{aligned}$$

## Compound classes

For a given “chemionic *ensemble*” $\epsilon$, the Hamiltonian of Eq. 7 is mainly specified through the quantities $\#chp(\epsilon )$ and $\#vtx(\epsilon )=\#chp(\epsilon )+1$, which enter into a parameter chart like Table 1. Parameter charts of “iso-chemionic” molecules (such as methane, ammonia, water, and hydrogen fluoride, for instance) thus refer to some topological relationship.

**Table 3:** Estimated numbers of anchor integrals for an oligomer A and its precursor B

$\textrm{A}_{\textrm{n}}{-}\textrm{B}_{\textrm{n}-1}$	$\textrm{n}$	“Overlaps”	“Kinetics”	“Attractions”	“Repulsions”
		$\#S^{(0)}$	$\#K^{(0)}$	$\#U^{(0)}$	$\#R^{(0)}$
$$\textrm{H}{-}\text {XV}_{3}$$		$$ 5^{2}$$	$$5^{2}$$	$$\le 5^{3}$$	$$5^{4}$$
$$\textrm{H}_{3}\textrm{C}\overset{\tau _0}{-}\text {XV}_{3}$$	0	$$ 8^{2}-4^{2}$$	$$8^{2}-4^{2} $$	$$\le (8^{3}-4^{3})$$	$$ 8^{4}-4^{4}$$
$\textrm{H}_{3}\textrm{C}\overset{\tau _{1}}{-}\text {CH}_{2}\text {XV}_{3}$	1	$11^{2}-7^{2}$	$11^{2}-7^{2}$	$\le (11^{3}-7^{3})$	$11^{4}-7^{4}$
$\textrm{H}_{3}\textrm{C}\overset{\tau _{2}}{-}\textrm{C}_{2}\textrm{H}_{4}\text {XV}_{3}$	2	$14^{2}-10^{2}$	$14^{2}-10^{2}$	$\le (14^{3}-10^{3})$	$14^{4}-10^{4}$
$$\textrm{H}_{3}\textrm{C}\overset{\tau _{3}}{-}\textrm{C}_{3}\textrm{H}_{6}\text {XV}_{3}$$	3	$$ 17^{2}-13^{2}$$	$$17^{2}-13^{2}$$	$$\le (17^{3}-13^{3})$$	$$17^{4}-13^{4}$$
$\textrm{H}_{3}\textrm{C}\overset{\tau _{4}}{-}\textrm{C}_{4}\textrm{H}_{8}\text {XV}_{3}$	4	$20^{2}-16^{2}$	$20^{2}-16^{2}$	$\le ( 20^{3}-16^{3})$	$20^{4}-16^{4}$

If the nuclear composition is even the same, family relations become apparently stronger. Table 2 indicates that (due to identical entries) normal alkanes, iso-alkanes, and neo-alkanes, primary, secondary, and tertiary alcohols, etc. form compound classes: their members only differ through $\{\textrm{Z}_{\epsilon C}^{prop},{C=1,\dots ,\#nuc}\}$ (i.e., the number and kind of nuclei of *ensemble* $\epsilon$) and another nuclear charge distribution, hence.

Considering chemionic intra-valence separations, on the other hand, distinctions of “functional groups” like $\text {CH}_{3}$ and $\text {CH}_{2}$ become evident. Most important, however, is the “chain bond” or “backbone bond” like $\text {CH}_{3}{-}\text {CH}_{2}$, which keeps its parameter chart constant as in Table 2. Parameter tables of identical form thus indicate a chemical relationship: molecular families or compound classes essentially show the same parameter table.

According to Primas and Müller-Herold, those quantum theoretical descriptions are preferable, which bring in some chemical evidence. For our purposes, Lorentz-relativistic kinematics, for instance, are useless; although being “better,” such a picture does not offer the desired systematic abstractions [81].

## Outlook and concluding remarks

There are at least two items, which could improve our model:

$$\circ $$
Making the vertex geometry more realistic through empirical bond lengths and angles [82]
$$\circ $$
Optimizing the few orbital exponents by nonlinear variation [77]

Its applicability could be enlarged by adding an Euler-Hückel treatment of:

$$\circ $$
Multiple bonds
$$\circ $$
Aromatic rings
$$\circ $$
Hydrogen bridges

In order to estimate the shape of proteins, condensed quantities for the 20 naturally occurring amino acids should enter into a data bank of fragments, together with the “peptide bond” [83]:

$$\begin{aligned} \dots -\underline{\textrm{N}}{\textrm{H}}{\overset{\phi }{-}}{\text {CH}}({-}{\textrm{R}}){\overset{\psi }{ -}}{\textrm{C}}({=}{\underline{\overline{\textrm{O}}}}){\overset{\omega }{ -}}\underline{\textrm{N}}{\textrm{H}}{-}\dots \end{aligned}$$

The side-chain R is a fragment of the naturally occurring $\alpha$-amino acids glycine (with R=H), alanine, serine, threonine, methionine, valine, leucine, isoleucine, phenylalanine, tyrosine, cysteine, aspartic acid, glutamic acid, arginine, lysine, histidine, tryptophan, asparagine, and glutamine. Proline has a five-membered ring instead of the $-\underline{\textrm{N}}{\textrm{H}-\text {CH}(-\textrm{R})}-$ moiety.

Similar considerations will be necessary for the conformation analysis of nucleic acids, which are alternant chains of phosphoric acids and pentose sugars, each substituted by a purin or pyrimidine base [84, 85]:

$$\begin{aligned} \dots {-}{\text {HPO}_{4}{-}\text {Pentose}({-}\text {Base}){-}\text {HPO}}_{4}{-}\dots \end{aligned}$$

$$\circ $$
Deoxyribonucleic acids (DNA) have the deoxiribose ($\textrm{C}_{5}\textrm{H}_{10}\textrm{O}_{4}$) as pentose-component, substituted by the purin bases adenine and guanine or the pyrimidines thymine and cytosine.
$$\circ $$
In ribonucleic acids (RNA), the deoxyribose is replaced by ribose ($\textrm{C}_{5}\textrm{H}_{10}\textrm{O}_{5}$), and the pyrimidine base thymine is replaced by uracil.

In contrast to polypeptides and proteins, DNA structures are better known. In 1953, after Chargaff had found the complementary pairing of adenine-thymine and guanine-cytosine, Watson and Crick proposed their famous “double helix model” [84, 85].

Protein geometries, on the other hand, are very hard to predict. For a given sequence of amino acids (called its “primary structure”), a couple of “secondary structures” may appear: $\alpha$-helices and $\beta$-strands, forming either parallel or anti-parallel sheets [83]. How such distinct domains fold into a more compact geometry is still an open question of biochemical sciences. “The general difficulties in obtaining protein structures using experimental techniques means that there is considerable interest in theoretical methods for predicting the three-dimensional structure of proteins from the amino acid sequence: this is often referred to as the *protein folding problem*” [60, 86–95].

While matrix dimensions can be kept constantly low during the described polymerization process, the evaluation of anchor integrals for each new torsion angle $\tau _{\textrm{n}}$ is still a “computational bottleneck” [40, 41] − especially of the “repulsions.” Due to the recursive nature of our Euler-Hückel picture, however, some of them were already stored during the precursor step of torsion optimization (see Table 3 for $\textrm{X}=\textrm{C},\textrm{N},\textrm{O},\textrm{F},\text {Si},\textrm{P},\textrm{S},\text {Cl}$ and their three vertices V):

Since

32 $$\begin{aligned} a^{2}-b^{2} =&(a-b)(a+b) \nonumber \\ a^{3} -b^{3} =&(a-b)(a^{2}+ab+b^{2}) \nonumber \\ a^{4}-b^{4} =&(a-b)(a^{3} + a^{2} b+ab^{2} + b^{3}) \end{aligned}$$

and the difference $(a-b)$ always is constant (namely 4), the task of “anchor integration” is considerably accelerated. “Linear scaling” [96] can be achieved, if we restrict ourselves to one- and two-center integrations, only.

Let us close this article with some fundamental considerations on quantum mechanics, in general. Physical theories can be characterized by an $*$-algebra of observables. Algebras of classical theories (like Newton’s mechanics, Maxwell’s electrodynamics, Clausius’ thermodynamics, and Einstein’s theory of relativity) are “commutative” (i.e., any two observables $\hat{A}$ an $\hat{B}$ interchange pairwise: $\hat{A}\hat{B}= \hat{B}\hat{A}$). The algebra of quantum mechanics, however, is “non-commutative” with incompatible observables: $\hat{A}\hat{B}\ne \hat{B}\hat{A}$. Different from the sharp values of classical observables, quantum theory deals with potential properties, which cannot be actualized simultaneously. For a thorough discussion of this topic, please consult the lectures and writings of Hans Primas, particularly his famous book of 1983, entitled “Chemistry, Quantum Mechanics and Reductionism” [18–25].

Following Ulrich Hoyer, on the other hand, Schrödinger’s (stationary and time-dependent) equations, for instance, can be derived by applying the statistical rules of Boltzmann’s distribution law. Alternatively, such a “Synthetic Quantum Theory” also emerges from Liouville’s theorem. It seems, that non-compatible properties of the molecular world can be readily explained by simple probability arguments. Let us just mention that such an “Ansatz” already shows the non-existence of $\text {He}_{2}, \text {Ne}_{2}$ and $\text {Be}_{2}$; for the diatomics $\textrm{H}_{2}, \text {Li}_{2}, \textrm{B}_{2}, \textrm{C}_{2}, \textrm{N}_{2}, \textrm{O}_{2}$ and $\textrm{F}_{2}$, it also leads to an estimation of dissociation energies; the bond length of $\textrm{H}_{2}$ is predicted as $\root 3 \of {4}$B, where B is the Bohr radius. According to Hoyer, pioneer quantum mechanics mainly suffers from a *“hýsteron-próteron”*: instead of proceeding with a belated probabilistic interpretation of the postulated wave function, one should start with purely statistical axioms from the very beginning [97–107].

Finally, we want to acknowledge for help and friendship. Moreover, we like to add three aphorisms of a home-attached philosopher, a cosmopolitan discoverer, and an expert for “life as work of art” [108], that might be encouraging for others, too.

“Ich stehe in der Einbildung, es sei zuweilen nicht unnütze, ein gewisses edles Vertrauen in seine eigenen Kräfte zu setzen. Eine Zuversicht von der Art belebet alle unsere Bemühungen, und erteilet ihnen einen gewissen Schwung, der der Untersuchung der Wahrheit sehr beförderlich ist.”

Immanuel Kant (1746) [109]

“Die Naturphilosophie kann den Fortschritten der empirischen Wissenschaften nie schädlich sein. Im Gegenteil, sie führt das Entdeckte auf Principien zurück, wie sie zugleich neue Entdeckungen begründet. Steht dabei eine Menschenklasse auf, welche es für bequemer hält, die Chemie durch die Kraft des Hirnes zu treiben, als sich die Hände zu benetzen, so ist das weder Ihre Schuld noch die der Naturphilosophie überhaupt. Darf man die Analysis verschreien, weil unsere Müller oft bessere Maschinen bauen als die, welche der Mathematiker berechnet hat ?”

Alexander von Humboldt (1. Februar 1805) an Friedrich Wilhelm Joseph Schelling [110]

“Wer will was Lebendigs erkennen und beschreiben, Sucht erst den Geist heraus zu treiben, Dann hat er die Teile in seiner Hand, Fehlt, leider ! nur das geistige Band. Encheiresin naturae nennt’s die Chemie, Spottet ihrer selbst und weiß nicht wie.”

Johann Wolfgang Goethe: “Faust I” (Ausgabe letzter Hand 1828) [111–113]

## Data Availability

Not applicable

## Appendix: Anchor data and first results

### A.1 Standard vertex geometries

Given the bond lengths $\lambda _{(\text {AV}_{\textrm{i}})}$, the dihedral angle $\delta =2\pi /3$ and with $\vartheta \overset{\text {def}}{=}\pi -\arccos \textstyle (-{1\over 3})$, the five vertex position vectors of the eight monofocal hydrids $\text {AV}_{4}$ read as in Table 4:

**Table 4:** Monofocal coordinates $(\textrm{A}=\textrm{C},\textrm{N},\textrm{O},\textrm{F},\text {Si},\textrm{P},\textrm{S},\text {Cl})$

$$\begin{array}{l}{\text {Anchor}} \\ {\text {coordinates}}\end{array}$$	$\textrm{a}_{0}$	$\textrm{a}_{1}$	$\textrm{a}_{2}$	$\textrm{a}_{3}$	$\textrm{a}_{4}$
*x*	0	$\sin (+\delta )\sin \vartheta \cdot \lambda _{(\text {AV}_{1})}$	$\sin (-\delta )\sin \vartheta \cdot \lambda _{(\text {AV}_{2})}$	0	0
*y*	0	$\cos (+\delta )\sin \vartheta \cdot \lambda _{(\text {AV}_{1})}$	$\cos (-\delta )\sin \vartheta \cdot \lambda _{(\text {AV}_{2})}$	$\sin \vartheta \cdot \lambda _{(\text {AV}_{3})}$	0
*z*	0	$-\cos \vartheta \cdot \lambda _{(\text {AV}_{1})}$	$-\cos \vartheta \cdot \lambda _{(\text {AV}_{2})}$	$-\cos \vartheta \cdot \lambda _{(\text {AV}_{3})}$	$+\lambda _{(\text {AV}_{4})}$

For A$=$C, reflecting the $\textbf{a}_{0}$, $\textbf{a}_{1}$, $\textbf{a}_{2}$, $\textbf{a}_{3}$ at the *xy*-plane and shifting them to the right (i.e., along the positive *z*-axis) by $\lambda _{(\text {CC})}$, we get another four vectors $\textbf{b}_{0}$, $\textbf{b}_{1}$, $\textbf{b}_{2}$, $\textbf{b}_{3}$, which all together define the “eclipsed” conformation of ethane $\text {CH}_{3}{-}\text {CH}_{3}$. For a “bifocal” methyl compound $\text {CH}_{3}{-}\text {XV}_{3}$, in general (where X = C, N, O, F, Si, P, S, Cl), we equivalently adopt the additional coordinates of Table 5.

**Table 5:** Additional coordinates for bifocal molecules

$$\begin{array}{l}{\text {Anchor}} \\ {\text {coordinates}}\end{array}$$	${\textrm{b}}_{0}$	${\textrm{b}}_{1}$	$\textrm{b}_{2}$	$\textrm{b}_{3}$
*x*	0	$\sin (+\delta )\sin \vartheta \cdot \lambda _{(\text {XV})}$	$\sin (-\delta )\sin \vartheta \cdot \lambda _{(\text {XV})}$	0
*y*	0	$\cos (+\delta )\sin \vartheta \cdot \lambda _{(\text {XV})}$	$\cos (-\delta )\sin \vartheta \cdot \lambda _{(\text {XV})}$	$\sin \vartheta \cdot \lambda _{(\text {XV})}$
*z*	$\lambda _{(\textrm{CX})}$	$\lambda _{(\text {CX})}+\cos \vartheta \cdot \lambda _{(\text {XV})}$	$\lambda _{(\text {CX})}+\cos \vartheta \cdot \lambda _{(\text {XV})}$	$\lambda _{(\text {CX})}+\cos \vartheta \cdot \lambda _{(\text {XV})}$

The extended chain $\textrm{H}_{3}\textrm{C}{\overset{\tau _{\textrm{n}}}{-}}(\text {CH}_{2})_{\textrm{n}}\text {XV}_3$ $({\textrm{n}=1,2,3,4,5,}$ $\dots )$ is also composed of two parts:

$\circ$: The constant methyl unit with four vertices $\{{\textbf{a}}_{i}|i=0,1,2,3\}$
$\circ$: The recursively extendable $(\text {CH}_{2})_{\textrm{n}}\text {XV}_{3}$ block with 3n+4 vertices $\{{\textbf{b}}^{\textrm{n}}_{i}|i=0,\dots ,3({\textrm{n}}{+}1)\}$.

Altogether, hence, we deal with $\{{\textbf{c}}^{\textrm{n}}_{i}|i=1,2,\dots ,3{\textrm{n}}{+}8\}$ vertices.

Defining a shift vector (with $\lambda \overset{\text {def}}{= } \lambda _{(\text {CC})}$), a rotation and a torsion matrix, respectively:

33 $$\begin{aligned} {\textbf{s}}(\lambda )&{=}&\left( \begin{array}{l} 0 \\ 0 \\ \lambda \end{array}\right) ;~ {\textbf{U}}(\vartheta ) {=} \left( \begin{array}{lcc} 1 &  0 &  0 \\ 0 &  +\cos (\vartheta ) &  +\sin (\vartheta ) \\ 0 &  -\sin (\vartheta ) &  +\cos (\vartheta ) \end{array}\right) ;~\nonumber \\ {\textbf{T}}(\tau _{n})&{=}&\left( \begin{array}{ccc} +\cos (\tau _{n}) &  -\sin (\tau _{n}) &  0 \\ +\sin (\tau _{n}) &  +\cos (\tau _{n}) &  0 \\ 0 &  0 &  1\end{array}\right) \end{aligned}$$

the bifocal geometries (i.e., of the ${\textrm{H}_{3}\textrm{C}}\overset{\tau _{0}}{-}{\text {XV}}_{3}$) and the polyfocal chain structures (i.e., of the ${\textrm{H}_{3}\textrm{C}}\overset{\tau _{\textrm{n}}}{-}({\text {CH}}_{2})_{\textrm{n}}{\text {XV}_{3}}$) respectfully, are mainly defined through the flexible set of $\textbf{b}_{i}^{\textrm{n}}$-vectors:

34 $$\begin{aligned}  &   \begin{array}{l} \textbf{b}_{0}^{0} = \textbf{T}(\tau _{0}) \textbf{b}_{0} \\ \textbf{b}_{1}^{0} = \textbf{T}(\tau _{0}) \textbf{b}_{1} \\ \textbf{b}_{2}^{0} = \textbf{T}(\tau _{0}) \textbf{b}_{2} \\ \textbf{b}_{3}^{0} = \textbf{T}(\tau _{0}) \textbf{b}_{3} \end{array} \quad ; \begin{aligned} \quad \textbf{b}_{0}^{\textrm{n}}&\!=\! \textbf{T}(\tau _{\textrm{n}}) {\textbf{U}}(\vartheta ) \left[ {\textbf{s}}(\lambda ) + {\textbf{a}}_{0} \right] \\ \quad {\textbf{b}}_{1}^{\textrm{n}}&\!=\! {\textbf{T}}(\tau _{\textrm{n}}) {\textbf{U}}(\vartheta ) \left[ {\textbf{s}}(\lambda ) + {\textbf{a}}_{1} \right] \\ \quad {\textbf{b}}_{2}^{\textrm{n}}&\!=\! {\textbf{T}}(\tau _{\textrm{n}}) {\textbf{U}}(\vartheta ) \left[ {\textbf{s}}(\lambda ) + {\textbf{a}}_{2} \right] \\ \quad {\textbf{b}}_{3}^{\textrm{n}}&\!=\! {\textbf{T}}(\tau _{\textrm{n}}) {\textbf{U}}(\vartheta ) \left[ {\textbf{s}}(\lambda ) + {\textbf{b}}_{0}^{\textrm{n}-1} \right] \\ \quad&\ \ \vdots \\ \quad {\textbf{b}}_{3\textrm{n}+3}^{\textrm{n}}&\!=\! {\textbf{T}}(\tau _{\textrm{n}}) {\textbf{U}}(\vartheta ) \left[ {\textbf{s}}(\lambda ) + {\textbf{b}}_{3\textrm{n}}^{\textrm{n}-1} \right] \end{aligned} \quad ;\nonumber \\  &   \begin{array}{l} {\textrm{n}=1,2,3,4,5,}\dots \end{array} \end{aligned}$$

The required torsion angle $\tau _{\textrm{n}}$ refers to the minimum of the potential curve $\mathcal {E}_{0}(\tau )$. In highly symmetric cases (as for “eclipsed” ethane (point group $D_{3v}$) and “staggered” ethane (point group $D_{3d}$)) energetic “degeneracies” will occur.

### A.2 Bond lengths

**Table 6:** Estimated bond lengths $\lambda _{(\text {XH})}$

*B* $\approx 0.529177249$ Å	$\lambda _{(\text {CH})}$	$\lambda _{(\text {NH})}$	$\lambda _{(\text {OH})}$	$\lambda _{(\text {FH})}$	$\lambda _{(\text {SiH})}$	$\lambda _{(\text {PH})}$	$\lambda _{(\text {SH})}$	$\lambda _{(\text {ClH})}$
Ångstr${\phi }$m (Å) [81]	1.096	1.021	0.965	0.917	1.486	1.417	1.327	1.275
Bohr (*B*)	2.071	1.929	1.824	1.733	2.808	2.678	2.508	2.409

See Tables 6 and 7. For the heteroatomic distances $\lambda _{(\text {AB})}$, we assume the following [114]:

35 $$\begin{aligned} \lambda _{(\text {AB})}:=(\lambda _{(\text {AA})}~+~\lambda _{(\text {BB})})/2 \end{aligned}$$

**Table 7:** Estimated bond lengths $\lambda _{(\text {XX})}$

*B* $\approx 0.529177249$ Å	$\lambda _{(\text {CC})}$	$\lambda _{(\text {NN})}$	$\lambda _{(\text {OO})}$	$\lambda _{(\text {FF})}$	$\lambda _{(\text {SiSi})}$	$\lambda _{(\text {PP})}$	$\lambda _{(\text {SS})}$	$\lambda _{(\text {ClCl})}$
Ångstr${\phi }$m (Å) [81]	1.531	1.449	1.452	1.412	2.327	2.219	2.055	1.988
Bohr (*B*)	2.893	2.738	2.744	2.668	4.397	4.193	3.883	3.757

### A.3 Optimized orbital exponents

**Table 8:** Vertex orbital exponents. Optimized by non-linear variation [77]

$\text {XV}_{4}$	$\text {CH}_{4}$	$\underline{\textrm{N}}\textrm{H}_{3}$	$\overline{\underline{\textrm{O}}}\textrm{H}_{2}$	$|\overline{\underline{\textrm{F}}}{\textrm{H}}$	$\text {SiH}_{4}$	$\underline{\textrm{P}}{\textrm{H}}_{3}$	$\overline{\underline{\textrm{S}}}\textrm{H}_{2}$	$|\overline{\underline{\text {Cl}}}{\textrm{H}}$
$\zeta ^{\textrm{X}}_{{\textrm{n}}^{val}\textrm{s}}$	3.135	7.059	4.929	4.143	3.513	7.631	5.101	4.919
$\zeta ^{\textrm{H}}_{1\textrm{s}}$	1.394	1.340	1.379	1.455	1.148	1.073	1.131	1.156

See Table 8.

### A.4 Proportional nuclear charges

Due to “chemionic separations” and “vertex condensations,” different nuclear charges enter into the diagonal and off-diagonal elements of attraction energy and their Fock matrices. Corresponding considerations hold for the nuclear repulsion energies. While in “diagonal” contributions $\textrm{V}_{\text {CC}}$ (and the corresponding $\mathcal {E}_{\epsilon (\text {dia})}^{nucl}$), $2\#chp$ chemions have to be proportionally distributed on a subset of vertices, “off-diagonal” terms $\textrm{V}_{\text {AB}}$ (and the corresponding $\mathcal {E}_{\epsilon (\text {off})}^{nucl}$) contribute very low nuclear charges—namely the proportional weight of only two chemions. See Tables 9 and 10.

**Table 9:** Nuclear charge distributions (“Diagonal”)

Lewis–Langmuir formula	$\#chp$	$\#vtx$	$\textrm{Z}^{prop}_{\text {love}}$	$\textrm{Z}^{prop}_{\textrm{H}}$	$$\begin{array}{l}\textrm{Z}^{prop}_{\textrm{C}}\\ \textrm{Z}^{prop}_{\textrm{Si}}\end{array}$$	$$\begin{array}{l}\textrm{Z}^{prop}_{\textrm{N}}\\ \textrm{Z}^{prop}_{\textrm{P}}\end{array}$$	$$\begin{array}{l}\textrm{Z}^{prop}_{\textrm{O}}\\ \textrm{Z}^{prop}_{\textrm{S}}\end{array}$$	$$\begin{array}{l}\textrm{Z}^{prop}_{\textrm{F}}\\ \textrm{Z}^{prop}_{\text {Cl}}\end{array}$$
$(\textrm{H}{-}\textrm{C}{=}\textrm{H}_{2}),(\textrm{X}{\equiv }\textrm{V}_{3})$	3	4	0	0.857	3.429	4.286	5.143	6.000
$(\textrm{C}{=}\textrm{H}_{2}){-}(\textrm{X}{\equiv }\textrm{V}_{3})$	6	7	0	0.923	3.692	4.615	5.538	6.462
$(\textrm{C}{=}\textrm{H}_{2}){-}(\textrm{C}{=}\textrm{H}_{2}){-}(\textrm{X}{\equiv }\textrm{V}_{3})$	9	10	0	0.947	3.789	4.737	5.684	6.632
$\vdots$	$\vdots$	$\vdots$	$\vdots$	$\vdots$	$\vdots$	$\vdots$	$\vdots$	$\vdots$

### A.5 Approximate three- and four-center repulsion integrals

In order to accelerate the described recursive procedure, we consider two additional techniques for the purpose of further simplification: an explicit and an implicit pathway.

#### A.5.1 Explicit integrations

For the “Coulomb-type three-center repulsions,” we write:

36 $$\begin{aligned} \begin{array}{l} R_{(a.b|c.c)}:=R_{(a.a|c.c)}{\dot{X}}_{(a.b)}^{\text {cou}}+R_{(b.b|c.c)}{\ddot{X}}_{(a.b)}^{\text {cou}}\\ R_{(a.a|c.d)}:=R_{(a.a|c.c)}{\dot{X}}_{(c.d)}^{\text {cou}}+R_{(a.a|d.d)}{\ddot{X}}_{(c.d)}^{\text {cou}} \end{array} \end{aligned}$$

For the “exchange-type three-center repulsions,” we write:

37 $$\begin{aligned} \begin{array}{l} R_{(a.c|b.c)}:=R_{(a.c|a.c)}{\dot{X}}_{(a.b)}^{\text {exc}}+R_{(b.c|b.c)}{\ddot{X}}_{(a.b)}^{\text {exc}}\\ R_{(a.c|a.d)}:=R_{(a.c|a.c)}{\dot{X}}_{(c.d)}^{\text {exc}}+R_{(a.d|a.d)}{\ddot{X}}_{(c.d)}^{\text {exc}} \end{array} \end{aligned}$$

With respect to the four-center integrals, in general, such distinctions cannot be made. Nevertheless, we consider four different approximations:

38 $$\begin{aligned} R_{(a.b|c.d)}:= &   {1\over 4}\left[ R_{(a.b|c.d)}^{\text {cou(a.b)}}+R_{(a.b|c.d)}^{\text {cou(c.d)}}+{\textstyle {1\over 2}}\left\{ R_{(a.c|b.d)}^{\text {exc(a.b)}}\right. \right. \nonumber \\  &   \quad \quad \!\!\!\!\left. \left. {+}R_{(a.d|c.b)}^{\text {exc(a.b)}}\right\} \!+\!{\textstyle {1\over 2}}\left\{ R_{(a.c|b.d)}^{\text {exc(c.d)}}\!{+}\!R_{(a.d|c.b)}^{\text {exc(c.d)}}\right\} \right] \end{aligned}$$

For the “Coulomb-type four-center repulsions,” we write:

39 $$\begin{aligned} R_{(a.b|c.d)}^{\text {cou(a.b)}}:= &   \left\{ \left[ R_{(a.a|c.c)}+R_{(a.a|d.d)}\right] {\dot{X}}_{(a.b)}^{\text {cou}}\right. \nonumber \\  &   ~~\left. + \left[ R_{(b.b|c.c)} + R_{(b.b|d.d)} \right] {\ddot{X}}_{(a.b)}^{\text {cou}} \right\} {Y}_{(a.b)}^{\text {cou}}\nonumber \\ R_{(a.b|c.d)}^{\text {cou(c.d)}}:= &   \left\{ \left[ R_{(a.a|c.c)} + R_{(b.b|c.c)} \right] {\dot{X}}_{(c.d)}^{\text {cou}}\right. \nonumber \\  &   ~~\left. + \left[ R_{(a.a|d.d)} + R_{(b.b|d.d)} \right] {\ddot{X}}_{(c.d)}^{\text {cou}} \right\} {Y}_{(c.d)}^{\text {cou}} \end{aligned}$$

For the “exchange-type four-center repulsions,” we write:

40 $$\begin{aligned} {\textstyle {1\over 2}} \left\{ \begin{array}{c} R_{(a.c|b.d)}^{\text {exc(a.b)}} \\ + \\ R_{(a.d|c.b)}^{\text {exc(a.b)}}\end{array} \right\}:= &   \left\{ \left[ R_{(a.c|a.c)} + R_{(a.d|a.d)} \right] {\dot{X}}_{(a.b)}^{\text {exc}}\right. \nonumber \\  &   ~\left. + \left[ R_{(b.c|b.c)} + R_{(b.d|b.d)} \right] {\ddot{X}}_{(a.b)}^{\text {exc}} \right\} {Y}_{(a.b)}^{\text {exc}}\nonumber \\ {\textstyle {1\over 2}} \left\{ \begin{array}{c} R_{(a.c|b.d)}^{\text {exc(c.d)}} \\ + \\ R_{(a.d|c.b)}^{\text {exc(c.d)}}\end{array} \right\} \!:= &   \! \left\{ \left[ R_{(a.c|a.c)} \!+\! R_{(b.c|b.c)} \right] {\dot{X}}_{(c.d)}^{\text {exc}}\right. \nonumber \\  &   \!\left. \!+\!\! \left[ R_{(a.d|a.d)} \!+\! R_{(b.d|b.d)} \!\right] {\ddot{X}}_{(c.d)}^{\text {exc}} \!\right\} {Y}_{(c.d)}^{\text {exc}} \end{aligned}$$

With the following substitutions, we get from Eq. (36_1_):

41 $$\begin{aligned} c=a:R_{(a.b|a.a)}=R_{(a.a|a.a)}{\dot{X}}_{(a.b)}^{\text {cou}}+R_{(b.b|a.a)}{\ddot{X}}_{(a.b)}^{\text {cou}}\nonumber \\ c=b:R_{(a.b|b.b)}=R_{(a.a|b.b)}{\dot{X}}_{(a.b)}^{\text {cou}}+R_{(b.b|b.b)}{\ddot{X}}_{(a.b)}^{\text {cou}} \nonumber \\ \left( \begin{array}{l} {\dot{X}}_{(a.b)}^{\text {cou}}\\ {\ddot{X}}_{(a.b)}^{\text {cou}} \end{array}\right) =\left( \begin{array}{l} R_{(a.a|a.a)}\qquad R_{(b.b|a.a)}\\ R_{(a.a|b.b)}\qquad R_{(b.b|b.b)} \end{array}\right) ^{-1} \left( \begin{array}{l} R_{(a.b|a.a)}\\ R_{(a.b|b.b)} \end{array}\right) \end{aligned}$$

**Table 10:** Nuclear charge distributions (“Off-Diagonal”)

Lewis–Langmuir formula	$\#chp$	$\#vtx$	$\textrm{Z}^{prop}_{\text {love}}$	$\textrm{Z}^{prop}_{\textrm{H}}$	$$\begin{array}{l}\textrm{Z}^{prop}_{\textrm{C}}\\ \textrm{Z}^{prop}_{\text {Si}}\end{array}$$	$$\begin{array}{l}\textrm{Z}^{prop}_{\textrm{N}}\\ \textrm{Z}^{prop}_{\textrm{P}}\end{array}$$	$$\begin{array}{l}\textrm{Z}^{prop}_{\textrm{O}}\\ \textrm{Z}^{prop}_{\textrm{S}}\end{array}$$	$$\begin{array}{l} \textrm{Z}^{prop}_{\textrm{F}}\\ \textrm{Z}^{prop}_{\text {Cl}}\end{array}$$
$(\text {HCH}_{2}{-}\text {XV}_{3})$	1	2	0	0.143	0.571	0.714	0.857	1.000
$(\text {HCH}_{2}{-}\text {CH}_{2}\text {XV}_{3})$	1	2	0	0.100	0.400	0.500	0.600	0.700
$(\text {HCH}_{2}{-}\text {CH}_{2}\text {CH}_{2}\text {XV}_{3})$	1	2	0	0.077	0.308	0.385	0.462	0.538
$\vdots$	$\vdots$	$\vdots$	$\vdots$	$\vdots$	$\vdots$	$\vdots$	$\vdots$	$\vdots$

**Table 11:** Singlet ground state energies, torsion angles, and rotational barriers (X=C,N,O,F)

$\mathrm Molecule$	^1^ $\mathcal {E}_{0}^{\text {min}}$	^1^$\tau ^{\text {min}}$(°)	^1^ $\mathcal {E}_{0}^{\text {max}}$	^1^$\tau ^{\text {max}}$(°)	^1^ $\mathcal {E}_{0}^{\Delta }$
$$\textrm{H}_{3}\textrm{C}{\overset{\tau _{0}}{-}}(\text {CH}_{2})_{0}\text {CH}_{3}$$	$$-6.477899 $$	240	$$-6.465910$$	300	0.011989
$$\textrm{H}_{3}\textrm{C}{\overset{\tau _{1}}{-}}(\text {CH}_{2})_{1}\text {CH}_{3}$$	$$-9.933752 $$	0	$$-9.864920$$	180	0.068831
$\textrm{H}_{3}\textrm{C}{\overset{\tau _{2}}{-}}(\text {CH}_{2})_{2}\text {CH}_{3}$	$-12.617469$	0	$-12.474409$	180	0.143060
$$\textrm{H}_{3}\textrm{C}{\overset{\tau _{3}}{-}}(\text {CH}_{2})_{3}\text {CH}_{3}$$	$$-14.935630$$	0	$$-14.771015 $$	180	0.164615
$\textrm{H}_{3}\textrm{C}{\overset{\tau _{4}}{-}}(\text {CH}_{2})_{4}\text {CH}_{3}$	$-18.372379$	180	$-18.043016$	244	0.329364
$$\textrm{H}_{3}\textrm{C}{\overset{\tau _{0}}{-}}(\text {CH}_{2})_{0}\text {NH}_{2}$$	$$-6.619495 $$	120	$$-6.610867$$	300	0.008628
$\textrm{H}_{3}\textrm{C}{\overset{\tau _{1}}{-}}(\text {CH}_{2})_{1}\text {NH}_{2}$	$-10.356975$	0	$-10.289834$	180	0.067141
$$\textrm{H}_{3}\textrm{C}{\overset{\tau _{2}}{-}}(\text {CH}_{2})_{2}\text {NH}_{2}$$	$$-13.066156 $$	0	$$-12.914883 $$	180	0.151273
$\textrm{H}_{3}\textrm{C}{\overset{\tau _{3}}{-}}(\text {CH}_{2})_{3}\text {NH}_{2}$	$-15.335027$	0	$-15.163633$	180	0.171394
$\textrm{H}_{3}\textrm{C}{\overset{\tau _{4}}{-}}(\text {CH}_{2})_{4}\text {NH}_{2}$	$-18.081551$	0	$-17.998870$	266	0.082681
$$\textrm{H}_{3}\textrm{C}{\overset{\tau _{0}}{-}}(\text {CH}_{2})_{0}\text {OH}$$	$$-9.096248 $$	120	$$-9.093689$$	300	0.002559
$\textrm{H}_{3}\textrm{C}{\overset{\tau _{1}}{-}}(\text {CH}_{2})_{1}\text {OH}$	$-12.360771$	359	$-12.299979$	178	0.060791
$$\textrm{H}_{3}\textrm{C}{\overset{\tau _{2}}{-}}(\text {CH}_{2})_{2}\text {OH}$$	$$ -14.454822$$	0	$$ -14.307775$$	180	0.147047
$$\textrm{H}_{3}\textrm{C}{\overset{\tau _{3}}{-}}(\text {CH}_{2})_{3}\text {OH}$$	$$ -16.386186 $$	0	$$ -16.217035$$	180	0.169151
$$\textrm{H}_{3}\textrm{C}{\overset{\tau _{4}}{-}}(\text {CH}_{2})_{4}\text {OH}$$	$$ -19.011485$$	178	$$ -18.864707 $$	112	0.146778
$$\textrm{H}_{3}\textrm{C}{\overset{\tau _{0}}{-}}(\text {CH}_{2})_{0}\textrm{F}$$	$$-11.369174 $$	120	$$ -11.368636$$	300	0.000538
$$\textrm{H}_{3}\textrm{C}{\overset{\tau _{1}}{-}}(\text {CH}_{2})_{1}\textrm{F}$$	$$-14.152210 $$	0	$$ -14.094922 $$	180	0.057288
$$\textrm{H}_{3}\textrm{C}{\overset{\tau _{2}}{-}}(\text {CH}_{2})_{2}\textrm{F}$$	$$ -15.763482$$	0	$$ -15.620912 $$	180	0.142570
$$\textrm{H}_{3}\textrm{C}{\overset{\tau _{3}}{-}}(\text {CH}_{2})_{3}\textrm{F}$$	$$ -17.405247$$	0	$$ -17.239494 $$	180	0.165753
$$\textrm{H}_{3}\textrm{C}{\overset{\tau _{4}}{-}}(\text {CH}_{2})_{4}\textrm{F}$$	$$-19.927925$$	180	$$-19.707767 $$	242	0.220158

**Table 12:** Singlet ground state energies, torsion angles, and rotational barriers (X=Si,P,S,Cl)

Molecule	^1^ $\mathcal {E}_{0}^{\text {min}}$	^1^$\tau ^{\text {min}}$(°)	^1^ $\mathcal {E}_{0}^{\text {max}}$	^1^$\tau ^{\text {max}}$(°)	^1^ $\mathcal {E}_{0}^{\Delta }$
$$\textrm{H}_{3}\textrm{C}{\overset{\tau _{0}}{-}} (\text {CH}_{2})_{0}\text {SiH}_{3}$$	$$-6.144395 $$	240	$$ -6.140868$$	300	0.003527
$$\textrm{H}_{3}\textrm{C}{\overset{\tau _{1}}{-}}(\text {CH}_{2})_{1}\text {SiH}_{3}$$	$$ -8.495787 $$	0	$$-8.423376 $$	180	0.072411
$\textrm{H}_{3}\textrm{C}{\overset{\tau _{2}}{-}}(\text {CH}_{2})_{2}\text {SiH}_{3}$	$-11.427476$	0	$-11.279735$	180	0.147741
$\textrm{H}_{3}\textrm{C}{\overset{\tau _{3}}{-}}(\text {CH}_{2})_{3}\text {SiH}_{3}$	$-13.963569$	0	$-13.796453$	180	0.167116
$$\textrm{H}_{3}\textrm{C}{\overset{\tau _{4}}{-}}(\text {CH}_{2})_{4}\text {SiH}_{3}$$	$$-17.039402 $$	180	$$-16.939083$$	250	0.100318
$$\textrm{H}_{3}\textrm{C}{\overset{\tau _{0}}{-}}(\text {CH}_{2})_{0}\text {PH}_{2}$$	$$-6.315448 $$	120	$$-6.310486 $$	300	0.004962
$\textrm{H}_{3}\textrm{C}{\overset{\tau _{1}}{-}}(\text {CH}_{2})_{1}\text {PH}_{2}$	$-9.267495$	0	$-9.199365$	180	0.068130
$$\textrm{H}_{3}\textrm{C}{\overset{\tau _{2}}{-}}(\text {CH}_{2})_{2}\text {PH}_{2}$$	$$ -12.128896$$	0	$$-11.977922$$	180	0.150974
$$\textrm{H}_{3}\textrm{C}{\overset{\tau _{3}}{-}}(\text {CH}_{2})_{3}\text {PH}_{2}$$	$$ -14.543336$$	0	$$-14.373232$$	180	0.170104
$\textrm{H}_{3}\textrm{C}{\overset{\tau _{4}}{-}}(\text {CH}_{2})_{4}\text {PH}_{2}$	$-17.291075$	0	$-17.193451$	205	0.097625
$$\textrm{H}_{3}\textrm{C}{\overset{\tau _{0}}{-}}(\text {CH}_{2})_{0}\text {SH}$$	$$ -8.491059 $$	120	$$-8.489176 $$	300	0.001884
$$\textrm{H}_{3}\textrm{C}{\overset{\tau _{1}}{-}}(\text {CH}_{2})_{1}\text {SH}$$	$$-11.418154$$	0	$$-11.361327 $$	178	0.056827
$\textrm{H}_{3}\textrm{C}{\overset{\tau _{2}}{-}}(\text {CH}_{2})_{2}\text {SH}$	$-13.636860$	0	$-13.496320$	180	0.140541
$$\textrm{H}_{3}\textrm{C}{\overset{\tau _{3}}{-}}(\text {CH}_{2})_{3}\text {SH}$$	$$-15.672831$$	0	$$-15.510166 $$	180	0.162665
$$\textrm{H}_{3}\textrm{C}{\overset{\tau _{4}}{-}}(\text {CH}_{2})_{4}\text {SH}$$	$$-18.297801$$	180	$$-18.169737 $$	127	0.128064
$$\textrm{H}_{3}\textrm{C}{\overset{\tau _{0}}{-}}(\text {CH}_{2})_{0}\text {Cl} $$	$$-10.829138 $$	120	$$ -10.828942 $$	60	0.000195
$$\textrm{H}_{3}\textrm{C}{\overset{\tau _{1}}{-}}(\text {CH}_{2})_{1}\text {Cl}$$	$$-13.331721 $$	0	$$-13.279587 $$	180	0.052134
$$\textrm{H}_{3}\textrm{C}{\overset{\tau _{2}}{-}}(\text {CH}_{2})_{2}\text {Cl}$$	$$-15.058243 $$	0	$$-14.922214 $$	180	0.136030
$\textrm{H}_{3}\textrm{C}{\overset{\tau _{3}}{-}}(\text {CH}_{2})_{3}\text {Cl}$	$-16.789003$	0	$-16.629432$	180	0.159572
$$\textrm{H}_{3}\textrm{C}{\overset{\tau _{4}}{-}}(\text {CH}_{2})_{4}\text {Cl}$$	$$-19.244582$$	180	$$-19.076360 $$	242	0.168222

**Table 13:** Triplet ground state energies, torsion angles, and rotational barriers (X=C,N,O,F)

$\mathrm Molecule$	^3^ $\mathcal {E}_{0}^{\text {min}}$	^3^$\tau ^{\text {min}}$(°)	^3^ $\mathcal {E}_{0}^{\text {max}}$	^3^$\tau ^{\text {max}}$(°)	^3^ $\mathcal {E}_{0}^{\Delta }$
$\textrm{H}_{3}\textrm{C}{\overset{\tau _{0}}{-}}(\text {CH}_{2})_{0}\text {CH}_{3}$	$-6.581994$	240	$-6.581832$	180	0.000162
$$\textrm{H}_{3}\textrm{C}{\overset{\tau _{1}}{-}}(\text {CH}_{2})_{1}\text {CH}_{3}$$	$$-8.415144 $$	0	$$-8.399894$$	183	0.015250
$$\textrm{H}_{3}\textrm{C}{\overset{\tau _{2}}{-}}(\text {CH}_{2})_{2}\text {CH}_{3}$$	$$-9.821553 $$	0	$$-9.776628$$	181	0.044925
$$\textrm{H}_{3}\textrm{C}{\overset{\tau _{3}}{-}}(\text {CH}_{2})_{3}\text {CH}_{3}$$	$$-11.055062 $$	0	$$-10.997966$$	180	0.057096
$$\textrm{H}_{3}\textrm{C}{\overset{\tau _{4}}{-}}(\text {CH}_{2})_{4}\text {CH}_{3}$$	$$-12.801917 $$	180	$$ -12.632568$$	243	0.169349
$$\textrm{H}_{3}\textrm{C}{\overset{\tau _{0}}{-}}(\text {CH}_{2})_{0}\text {NH}_{2} $$	$$ -6.493055$$	120	$$-6.492776 $$	298	0.000280
$\textrm{H}_{3}\textrm{C}{\overset{\tau _{1}}{-}}(\text {CH}_{2})_{1}\text {NH}_{2}$	$-8.678021$	120	$-8.667070$	304	0.010951
$$\textrm{H}_{3}\textrm{C}{\overset{\tau _{2}}{-}}(\text {CH}_{2})_{2}\text {NH}_{2}$$	$$-10.070885$$	0	$$-10.027990 $$	299	0.042895
$$\textrm{H}_{3}\textrm{C}{\overset{\tau _{3}}{-}}(\text {CH}_{2})_{3}\text {NH}_{2}$$	$$ -11.269107$$	0	$$-11.214333$$	180	0.054774
$\textrm{H}_{3}\textrm{C}{\overset{\tau _{4}}{-}}(\text {CH}_{2})_{4}\text {NH}_{2}$	$-12.669951$	0	$-12.640758$	267	0.029193
$$\textrm{H}_{3}\textrm{C}{\overset{\tau _{0}}{-}}(\text {CH}_{2})_{0}\text {OH}$$	$$-8.185327 $$	121	$$-8.185257 $$	312	0.000069
$\textrm{H}_{3}\textrm{C}{\overset{\tau _{1}}{-}}(\text {CH}_{2})_{1}\text {OH}$	$-9.759177$	120	$-9.747680$	297	0.011498
$$\textrm{H}_{3}\textrm{C}{\overset{\tau _{2}}{-}}(\text {CH}_{2})_{2}\text {OH} $$	$$-10.802832$$	0	$$-10.758532 $$	299	0.044300
$$\textrm{H}_{3}\textrm{C}{\overset{\tau _{3}}{-}}(\text {CH}_{2})_{3}\text {OH} $$	$$-11.812668 $$	0	$$-11.757137 $$	181	0.055532
$$\textrm{H}_{3}\textrm{C}{\overset{\tau _{4}}{-}}(\text {CH}_{2})_{4}\text {OH}$$	$$ -13.151216 $$	178	$$-13.085222 $$	109	0.065995
$$\textrm{H}_{3}\textrm{C}{\overset{\tau _{0}}{-}}(\text {CH}_{2})_{0}\text {F}$$	$$-9.403153 $$	300	$$-9.403084 $$	240	0.000069
$$\textrm{H}_{3}\textrm{C}{\overset{\tau _{1}}{-}}(\text {CH}_{2})_{1}\text {F}$$	$$ -10.723688$$	120	$$ -10.712088$$	301	0.011600
$$\textrm{H}_{3}\textrm{C}{\overset{\tau _{2}}{-}}(\text {CH}_{2})_{2}\text {F}$$	$$-11.505566 $$	0	$$-11.460910$$	299	0.044655
$$\textrm{H}_{3}\textrm{C}{\overset{\tau _{3}}{-}}(\text {CH}_{2})_{3}\text {F}$$	$$-12.353479 $$	0	$$-12.298000 $$	180	0.055479
$$\textrm{H}_{3}\textrm{C}{\overset{\tau _{4}}{-}}(\text {CH}_{2})_{4}\text {F}$$	$$-13.631257$$	180	$$-13.526904 $$	241	0.104352

**Table 14:** Triplet ground state energies, torsion angles, and rotational barriers (X=Si,P,S,Cl)

$\mathrm Molecule$	^3^ ${\mathcal {E}}_{0}^{\text {min}}$	^3^$\tau ^{\text {min}}$(°)	^3^ $\mathcal {E}_{0}^{\text {max}}$	^3^$\tau ^{\text {max}}$(°)	^3^ $\mathcal {E}_{0}^{\Delta }$
$$\textrm{H}_{3}\textrm{C}{\overset{\tau _{0}}{-}}(\text {CH}_{2})_{0}\text {SiH}_{3}$$	$$-5.649347$$	300	$$-5.649176 $$	240	0.000171
$$\textrm{H}_{3}\textrm{C}{\overset{\tau _{1}}{-}}(\text {CH}_{2})_{1}\text {SiH}_{3}$$	$$-7.602432 $$	120	$$-7.593195$$	305	0.009236
$$\textrm{H}_{3}\textrm{C}{\overset{\tau _{2}}{-}}(\text {CH}_{2})_{2}\text {SiH}_{3}$$	$$-9.198994 $$	0	$$ -9.161413 $$	299	0.037581
$$\textrm{H}_{3}\textrm{C}{\overset{\tau _{3}}{-}}(\text {CH}_{2})_{3}\text {SiH}_{3}$$	$$-10.558306$$	0	$$-10.506992 $$	180	0.051315
$$\textrm{H}_{3}\textrm{C}{\overset{\tau _{4}}{-}}(\text {CH}_{2})_{4}\text {SiH}_{3}$$	$$-12.135837$$	180	$$-12.087637 $$	250	0.048200
$\textrm{H}_{3}\textrm{C}{\overset{\tau _{0}}{-}}(\text {CH}_{2})_{0}\text {PH}_{2}$	$-6.164004$	63	$-6.163762$	241	0.000242
$\textrm{H}_{3}\textrm{C}{\overset{\tau _{1}}{-}}(\text {CH}_{2})_{1}\text {PH}_{2}$	$-8.089615$	120	$-8.080204$	306	0.009411
$\textrm{H}_{3}\textrm{C}{\overset{\tau _{2}}{-}}(\text {CH}_{2})_{2}\text {PH}_{2}$	$-9.588297$	0	$-9.547950$	299	0.040347
$\textrm{H}_{3}\textrm{C}{\overset{\tau _{3}}{-}}(\text {CH}_{2})_{3}\text {PH}_{2}$	$-10.868121$	0	$-10.815259$	180	0.052862
$$\textrm{H}_{3}\textrm{C}{\overset{\tau _{4}}{-}}(\text {CH}_{2})_{4}\text {PH}_{2}$$	$$ -12.280396$$	0	$$-12.244769 $$	204	0.035626
$$\textrm{H}_{3}\textrm{C}{\overset{\tau _{0}}{-}}(\text {CH}_{2})_{0}\text {SH}$$	$$-7.884158$$	179	$$ -7.884031$$	0	0.000128
$$\textrm{H}_{3}\textrm{C}{\overset{\tau _{1}}{-}}(\text {CH}_{2})_{1}\text {SH}$$	$$ -9.280606$$	120	$$-9.271048$$	297	0.009558
$$\textrm{H}_{3}\textrm{C}{\overset{\tau _{2}}{-}}(\text {CH}_{2})_{2}\text {SH}$$	$$-10.395408$$	0	$$ -10.354947$$	299	0.040461
$$\textrm{H}_{3}\textrm{C}{\overset{\tau _{3}}{-}}(\text {CH}_{2})_{3}\text {SH}$$	$$-11.460384$$	0	$$ -11.408486$$	180	0.051897
$$\textrm{H}_{3}\textrm{C}{\overset{\tau _{4}}{-}}(\text {CH}_{2})_{4}\text {SH}$$	$$ -12.802698$$	180	$$ -12.745900 $$	252	0.056798
$$\textrm{H}_{3}\textrm{C}{\overset{\tau _{0}}{-}}(\text {CH}_{2})_{0}\text {Cl}$$	$$-9.157441$$	300	$$-9.157389 $$	0	0.000052
$\textrm{H}_{3}\textrm{C}{\overset{\tau _{1}}{-}}(\text {CH}_{2})_{1}\text {Cl}$	$-10.315734$	120	$-10.306018$	301	0.009716
$$\textrm{H}_{3}\textrm{C}{\overset{\tau _{2}}{-}}(\text {CH}_{2})_{2}\text {Cl}$$	$$-11.157493 $$	0	$$-11.116265 $$	299	0.041228
$$\textrm{H}_{3}\textrm{C}{\overset{\tau _{3}}{-}}(\text {CH}_{2})_{3}\text {Cl}$$	$$-12.050235$$	0	$$-11.997984 $$	299	0.052252
$$\textrm{H}_{3}\textrm{C}{\overset{\tau _{4}}{-}}(\text {CH}_{2})_{4}\text {Cl}$$	$$-13.296249$$	180	$$ -13.220460$$	241	0.075789

With the following substitutions, we get from Eq. (37_1_):

42 $$\begin{aligned} c=a:R_{(a.b|a.a)}=R_{(a.a|a.a)}{\dot{X}}_{(a.b)}^{\text {exc}}+R_{(b.a|b.a)}{\ddot{X}}_{(a.b)}^{\text {exc}} \nonumber \\ c=b:R_{(a.b|b.b)}=R_{(a.b|a.b)}{\dot{X}}_{(a.b)}^{\text {exc}}+R_{(b.b|b.b)}{\ddot{X}}_{(a.b)}^{\text {exc}}\nonumber \\ \left( \begin{array}{l} {\dot{X}}_{(a.b)}^{\text {exc}}\\ {\ddot{X}}_{(a.b)}^{\text {exc}}\end{array}\right) = \left( \begin{array}{l} R_{(a.a|a.a)}\qquad R_{(b.a|b.a)}\\ R_{(a.b|a.b)}\qquad R_{(b.b|b.b)}\end{array}\right) ^{-1} \left( \begin{array}{l} R_{(a.b|a.a)}\\ R_{(a.b|b.b)} \end{array}\right) \end{aligned}$$

With the substitution $c.d=a.b$, we get from Eq. (39_1_):

43 $$\begin{aligned} R_{(a.b|a.b)}= &   \left\{ \left[ R_{(a.a|a.a)} + R_{(a.a|b.b)} \right] {\dot{X}}_{(a.b)}^{\text {cou}}\right. \nonumber \\  &   ~\left. + \left[ R_{(b.b|a.a)} + R_{(b.b|b.b)} \right] {\ddot{X}}_{(a.b)}^{\text {cou}} \right\} {Y}_{(a.b)}^{\text {cou}} \nonumber \\ {Y}_{(a.b)}^{\text {cou}}= &   \left\{ \left[ R_{(a.a|a.a)} + R_{(a.a|b.b)} \right] {\dot{X}}_{(a.b)}^{\text {cou}}\right. \nonumber \\  &   \left. \!+\! \left[ \! R_{(b.b|a.a)} \!+\! R_{(b.b|b.b)} \!\right] {\ddot{X}}_{(a.b)}^{\text {cou}} \!\right\} ^{-1} R_{(a.b|a.b)} \end{aligned}$$

With the substitution $c.d=a.b$, we get from Eq. (40_1_):

44 $$\begin{aligned}  &   {\textstyle {1\over 2}} \left\{ \begin{array}{c} R_{(a.a|b.b)} \\ + \\ R_{(a.b|a.b)}\end{array}\right\} = \left\{ \left[ R_{(a.a|a.a)}+R_{(a.b|a.b)}\right] {\dot{X}}_{(a.b)}^{\text {exc}}\right. \nonumber \\  &   \qquad \qquad \qquad \qquad \quad \left. +\left[ R_{(b.a|b.a)}+R_{(b.b|b.b)}\right] {\ddot{X}}_{(a.b)}^{\text {exc}}\right\} {Y}_{(a.b)}^{\text {exc}}\nonumber \\  &   {Y}_{(a.b)}^{\text {exc}}=\left\{ \left[ R_{(a.a|a.a)}+R_{(a.b|a.b)}\right] {\dot{X}}_{(a.b)}^{\text {exc}}\right. \nonumber \\  &   \quad \left. +\left[ R_{(b.a|b.a)}+R_{(b.b|b.b)}\right] {\ddot{X}}_{(a.b)}^{\text {exc}}\right\} ^{-1} \left\{ \begin{array}{c} R_{(a.a|b.b)} \\ + \\ R_{(a.b|a.b)}\end{array}\right\} {\textstyle {1\over 2}} \end{aligned}$$

#### A.5.2 Implicit integrations

In Roothaan’s “Restricted Hartree-Fock” theory (RHF) [40, 41], a couple of two-chemion repulsion integrals $R_{(a.b|c.d)}$ occur. Recall that

45 $$\begin{aligned} F_{\epsilon (m.n)}&\overset{\mathrm{~def}}{=}\  &H_{\epsilon (m.n)}+W_{\epsilon (m.n)}^{\text {cou(I)}}+W_{\epsilon (m.n)}^{\text {cou(II)}}\nonumber \\  &   -{\textstyle {1\over 2}}W_{\epsilon (m.n)}^{\text {exc(I)}}-{\textstyle {1\over 2}}W_{\epsilon (m.n)}^{\text {exc(II)}} \end{aligned}$$

where we separated the off-diagonal two-chemion part into a three-index and a four-index contribution:

46 $$\begin{aligned}  &   W_{\epsilon (m.n)}^{\text {cou(I)}}\overset{~\text {def}}{= }\sum _{p=1}^{\underset{(\epsilon )}{\#vtx}}R_{(m.n|p.p)}D_{\epsilon (p.p)}\quad ;\nonumber \\  &   W_{\epsilon (m.n)}^{\text {cou(II)}}\overset{~\text {def}}{= }\sum _{p=1}^{\underset{(\epsilon )}{\#vtx}}\sum _{q\ne p}^{\underset{(\epsilon )}{\#vtx}}R_{(m.n|p.q)}D_{\epsilon (p.q)} \end{aligned}$$

47 $$\begin{aligned}  &   W_{\epsilon (m.n)}^{\text {exc(I)}} \overset{~\text {def}}{= } \sum _{p=1}^{\underset{(\epsilon )}{\#vtx}} {\textstyle {1\over 2}} \left\{ \begin{array}{l} R_{(m.p|n.p)}\\ \qquad + \\ R_{(m.p|p.n)} \end{array} \right\} D_{\epsilon (p.p)}~;\nonumber \\  &   W_{\epsilon (m.n)}^{\text {exc(II)}}\overset{~\text {def}}{= }\sum _{p=1}^{\underset{(\epsilon )}{\#vtx}}\sum _{q\ne p}^{\underset{(\epsilon )}{\#vtx}}{\textstyle {1\over 2}} \left\{ \begin{array}{l} R_{(m.p|n.q)}\\ \qquad + \\ R_{(m.q|p.n)}\end{array}\right\} D_{\epsilon (p.q)} \end{aligned}$$

Using the formalism of the previous section, we get for the off-diagonal terms the following:

48 $$\begin{aligned} W_{\epsilon (m.n)}^{\text {cou(I)}}= &   {\dot{X}}_{(m.n)}^{\text {cou}}\underbrace{\sum _{p=1}^{\underset{(\epsilon )}{\#vtx}}R_{(m.m|p.p)}D_{\epsilon (p.p)}}_{=W_{\epsilon (m.m)}^{\text {cou(I)}}}\nonumber \\  &   +{\ddot{X}}_{(m.n)}^{\text {cou}}\underbrace{\sum _{p=1}^{\underset{(\epsilon )}{\#vtx}}R_{(n.n|p.p)}D_{\epsilon (p.p)}}_{=W_{\epsilon (n.n)}^{\text {cou(I)}}} \end{aligned}$$

49 $$\begin{aligned} W_{\epsilon (m.n)}^{\text {exc(I)}}= &   {\dot{X}}_{(m.n)}^{\text {exc}} \underbrace{ \sum _{p=1}^{\underset{(\epsilon )}{\#vtx}} {\textstyle {1\over 2}} \left\{ \begin{array}{l} R_{(m.p|m.p)}\\ \qquad + \\ R_{(m.p|p.m)}\end{array} \right\} D_{\epsilon (p.p)} }_{ =W_{\epsilon (m.m)}^{\text {exc(I)}} }\nonumber \\  &   + {\ddot{X}}_{(m.n)}^{\text {exc}} \underbrace{ \sum _{p=1}^{\underset{(\epsilon )}{\#vtx}} {\textstyle {1\over 2}} \left\{ \begin{array}{l} R_{(n.p|n.p)}\\ \qquad + \\ R_{(n.p|p.n)}\end{array} \right\} D_{\epsilon (p.p)} }_{ =W_{\epsilon (n.n)}^{\text {exc(I)}} } \end{aligned}$$

50 $$\begin{aligned} W_{\epsilon (m.n)}^{\text {cou(II)}}= &   {Y}_{(m.n)}^{\text {cou}}\underbrace{\sum _{p=1}^{\underset{(\epsilon )}{\#vtx}}\sum _{q\ne p}^{\underset{(\epsilon )}{\#vtx}}R_{(m.m|p.q)}D_{\epsilon (p.q)}}_{=W_{\epsilon (m.m)}^{\text {cou(II)}}}\nonumber \\  &   +{Y}_{(m.n)}^{\text {cou}}\underbrace{\sum _{p=1}^{\underset{(\epsilon )}{\#vtx}}\sum _{q\ne p}^{\underset{(\epsilon )}{\#vtx}}R_{(n.n|p.q)}D_{\epsilon (p.q)}}_{=W_{\epsilon (n.n)}^{\text {cou(II)}}} \end{aligned}$$

51 $$\begin{aligned} W_{\epsilon (m.n)}^{\text {exc(II)}}&{=}&{Y}_{(m.n)}^{\text {exc}} \underbrace{ \sum _{p=1}^{\underset{(\epsilon )}{\#vtx}} \sum _{q\ne p}^{\underset{(\epsilon )}{\#vtx}} {\textstyle {1\over 2}} \left\{ \begin{array}{l} R_{(m.p|m.q)}\\ \qquad + \\ R_{(m.q|p.m)}\end{array} \right\} D_{\epsilon (p.q)} }_{ =W_{\epsilon (m.m)}^{\text {exc(II)}} }\nonumber \\  &   + {Y}_{(m.n)}^{\text {exc}} \underbrace{ \sum _{p=1}^{\underset{(\epsilon )}{\#vtx}} \sum _{q\ne p}^{\underset{(\epsilon )}{\#vtx}} {\textstyle {1\over 2}} \left\{ \begin{array}{l} R_{(n.p|n.q)}\\ \qquad + \\ R_{(n.q|p.n)}\end{array} \right\} D_{\epsilon (p.q)} }_{ =W_{\epsilon (n.n)}^{\text {exc(II)}} } \end{aligned}$$

Having the same form as the Wolfsberg-Helmholz formula [115–118], the Eqs. 48–51 lay the non-empirical foundations for Roald Hoffmann’s “Extended Hückel Theory” [115–118].

The diagonal elements $W_{\epsilon (m.m)}^{\text {cou(II)}}$ and $W_{\epsilon (m.m)}^{\text {exc(II)}}$, which also contain two-chemion three-index interactions, reduce to a compact form, too:

52 $$\begin{aligned} W_{\epsilon (m.m)}^{\text {cou(II)}}= &   \sum _{p=1}^{\underset{(\epsilon )}{\#vtx}} R_{(m.m|p.p)} \underbrace{ \sum _{q\ne p}^{\underset{(\epsilon )}{\#vtx}}{\dot{X}}_{(p.q)}^\textrm{cou}D_{\epsilon (p.q)}}_{\overset{~\text {def}}{= }{\dot{D}}_{\epsilon (p.p)}^{\text {cou}}}\nonumber \\  &   +\sum _{q=1}^{\underset{(\epsilon )}{\#vtx}}R_{(m.m|q.q)}\underbrace{\sum _{p\ne q}^{\underset{(\epsilon )}{\#vtx}}{\ddot{X}}_{(p.q)}^{\text {cou}}D_{\epsilon (p.q)}}_{\overset{~\text {def}}{= }{\ddot{D}}_{\epsilon (q.q)}^{\text {cou}}} \end{aligned}$$

53 $$\begin{aligned} W_{\epsilon (m.m)}^{\text {exc(II)}}&{=}&\sum _{p=1}^{\underset{(\epsilon )}{\#vtx}}{\textstyle {1\over 2}} \left\{ \begin{array}{l} R_{(m.p|m.p)}\\ \qquad + \\ R_{(m.p|p.m)} \end{array} \right\} \underbrace{ \sum _{q\ne p}^{\underset{(\epsilon )}{\#vtx}} {\dot{X}}_{(p.q)}^{\text {exc}} D_{\epsilon (p.q)} }_{ \overset{~\text {def}}{= } {\dot{D}}_{\epsilon (p.p)}^{\text {exc}} }\nonumber \\  &   + \sum _{q=1}^{\underset{(\epsilon )}{\#vtx}} {\textstyle {1\over 2}} \left\{ \begin{array}{l} R_{(m.q|m.q)}\\ \qquad + \\ R_{(m.q|q.m)} \end{array} \right\} \underbrace{ \sum _{p\ne q}^{\underset{(\epsilon )}{\#vtx}} {\ddot{X}}_{(p.q)}^{\text {exc}} D_{\epsilon (p.q)} }_{ \overset{\mathrm{~def}}{= } {\ddot{D}}_{\epsilon (q.q)}^\textrm{exc} } \end{aligned}$$

Due to their ability of leading back repulsive multi-center interactions on only one- and two-center integrations, the above expressions yield considerable accelerations. Their use will be another important step towards a “linear scaling” of our recursive algorithm [96].

### A.6 Polyfocal ground state energies, torsion angles, and rotational barriers

All the “Born-Oppenheimer Energy Functions (BOEF)” discussed here are one-dimensional singlet and triplet state energies (i.e., potential curves). Mainly interesting

$\circ$: are their minimum $\mathcal {E}_{0}^{\text {min}}$ and the corresponding torsion angle $\tau ^{\text {min}}$
$\circ$: are their maximum $\mathcal {E}_{0}^{\text {max}}$ and the corresponding torsion angle $\tau ^{\text {max}}$
$\circ$: and the difference $\mathcal {E}_{0}^{\Delta }$, which corresponds to a “barrier of internal rotation.”

#### A.6.1 Singlet ground state energies

See Tables 11 and 12. Note, for instance, that the “eclipsed” conformation of ethane (with ^1^$\tau _{0}^{\text {min}}=0^{\circ },120^{\circ },240^{\circ }$) is predicted to be 0.011989 a.u. ($\approx$ 7.523 kcal/mole) more stable than the “staggered” one (with ^1^$\tau _{0}^{\text {max}}=60^{\circ },180^{\circ },300^{\circ }$). The same observation holds for $\textrm{H}_{3}\textrm{C}{\overset{^{1}\tau _0}{-}}(\text {CH}_{2})_{0}\text {SiH}_{3}$, $\textrm{H}_{3}\textrm{C}{\overset{^{1}\tau _0}{-}} (\text {CH}_{2})_{0}\text {NH}_{2}$ and $\textrm{H}_{3}\textrm{C}{\overset{^1\tau _0}{-}}(\text {CH}_{2})_{0}\text {PH}_{2}$, $\textrm{H}_{3}\textrm{C}{\overset{^{1}\tau _{0}}{-}} (\text {CH}_{2})_{0}\text {OH}$ and $\textrm{H}_{3}\textrm{C}{\overset{^{1}\tau _{0}}{-}} (\text {CH}_{2})_{0}$ $\text {SH}$. Rotational barriers of methylfluoride and -chloride, however, are an obvious arfifact of our model.

#### A.6.2 Triplet ground state energies

See Tables 13 and 14. Looking at the energies of the triplet ground state, sometimes, the observation can be made, that the “eclipsed” conformation (with ^3^$\tau _{0}^{\text {min}}=0^{\circ },120^{\circ },240^{\circ }$) is predicted to be less stable than the “staggered” one (with ^3^$\tau _{0}^{\text {max}}=60^{\circ },180^{\circ },300^{\circ }$); namely for $\textrm{H}_{3}\textrm{C}{\overset{^{3}\tau _{0}}{-}}{(\text {CH}_{2})_{0}\text {SiH}_{3}}$, $\textrm{H}_{3}\textrm{C}{\overset{^{3}\tau _{0}}{-}}{(\text {CH}_{2})_{0}\text {PH}_{2}}$, $\textrm{H}_{3}\textrm{C}{\overset{^{3}\tau _{0}}{-}}$ ${(\text {CH}_{2})_{0}\text {SH}}$, as well as for methylfluoride and -chloride. Rotational barriers of all these cases, however, are very low (less than 0.1 kcal/mole).
