# Correction to: Quantum chemical “Aufbau” principles: how to estimate the shape of highly flexible (bio-)polymers? A recursively extendable “chemion picture” of Euler-Hückel-type

**DOI:** 10.1007/s00894-024-05864-w

**Published:** 2024-02-23

**Authors:** Wolfhard H. G. Koch

**Affiliations:** 1 Facultad de Estudios Superiores Zaragoza, Universidad Nacional Autónoma de México, Mexico City, Mexico; 2 Institut für Physikalische und Theoretische Chemie, Universität Tübingen, Tübingen, Germany

**Correction to: Journal of Molecular Modeling (2024) 30:47**

10.1007/s00894-023-05807-x

I realized, that many of my amendments and corrections, which I sent to the Production team have not been taken into consideration. In particular, all my notes including literal citations have been totally skipped, as well as some German quotations; so that the article became incomplete and less understandable.

Now, even some additional and unnecessary mistakes came in:

• The list of keywords has been shortened. “Recursively extendable ‘Aufbau’ principles (REAP) and polymerizations” is missing.

• Please remove the slash and write “methylamine” instead of “methyl/amine”.

• On page 7, 9, 14, and 15, suddenly a couple of huge gaps appeared.

• Three German quotes (Kant, Humboldt, Goethe), which close the article, have been fully ignored. Without them, the corresponding references [98–101] disappeared from the text; which makes no sense, of course. Please insert them literally before continuing with the Appendix. Studying a “Springer” publication the reader sometimes must be prepared for finding German vocabulary.

The original article has been corrected.

